# The microbiota-gut-brain axis participates in chronic cerebral hypoperfusion by disrupting the metabolism of short-chain fatty acids

**DOI:** 10.1186/s40168-022-01255-6

**Published:** 2022-04-17

**Authors:** Weiping Xiao, Jiabin Su, Xinjie Gao, Heng Yang, Ruiyuan Weng, Wei Ni, Yuxiang Gu

**Affiliations:** 1 Department of Neurosurgery, Huashan Hospital, Fudan University, Shanghai, 200040 China; 2 Institute of Neurosurgery, Fudan University, Shanghai, 200052 China; 3 Shanghai Key Laboratory of Brain Function and Restoration and Neural Regeneration, Shanghai, 200052 China

**Keywords:** Chronic cerebral hypoperfusion, Gut dysbiosis, Gut microbiota, Fecal microbiota transplantation, Short-chain fatty acids

## Abstract

**Background:**

Chronic cerebral hypoperfusion (CCH) underlies secondary brain injury following certain metabolic disorders and central nervous system (CNS) diseases. Dysregulation of the microbiota-gut-brain axis can exacerbate various CNS disorders through aberrantly expressed metabolites such as short-chain fatty acids (SCFAs). Yet, its relationship with CCH remains to be demonstrated. And if so, it is of interest to explore whether restoring gut microbiota to maintain SCFA metabolism could protect against CCH.

**Results:**

Rats subjected to bilateral common carotid artery occlusion (BCCAO) as a model of CCH exhibited cognitive impairment, depressive-like behaviors, decreased gut motility, and compromised gut barrier functions. The 16S ribosomal RNA gene sequencing revealed an abnormal gut microbiota profile and decreased relative abundance of some representative SCFA producers, with the decreased hippocampal SCFAs as the further evidence. Using fecal microbiota transplantation (FMT), rats recolonized with a balanced gut microbiome acquired a higher level of hippocampal SCFAs, as well as decreased neuroinflammation when exposed to lipopolysaccharide. Healthy FMT promoted gut motility and gut barrier functions, and improved cognitive decline and depressive-like behaviors by inhibiting hippocampal neuronal apoptosis in BCCAO rats. Long-term SCFA supplementation further confirmed its neuroprotective effect in terms of relieving inflammatory response and hippocampal neuronal apoptosis following BCCAO.

**Conclusion:**

Our results demonstrate that modulating the gut microbiome via FMT can ameliorate BCCAO-induced gut dysbiosis, cognitive decline, and depressive-like behaviors, possibly by enhancing the relative abundance of SCFA-producing floras and subsequently increasing SCFA levels.

**Video abstract**

**Supplementary Information:**

The online version contains supplementary material available at 10.1186/s40168-022-01255-6.

## Background

Cerebrovascular diseases can damage brain vasculature and functional regions, leaving patients with physical disabilities, cognitive impairment, or depressive-like behaviors. Often coexisting with Alzheimer’s disease (AD) [1, 2], vascular dementia (VaD) is the second most common subtype of dementia in elderly individuals. It is the most serious stage and form of vascular cognitive impairment (VCI), and is linked with problems in reasoning, planning, judging, and memorizing [3, 4]. VaD is estimated to make up approximately 15 to 20% of dementia cases in North America and Europe, and a higher proportion, up to 30%, in Asia [5–8]. Chronic cerebral hypoperfusion (CCH) is considered to be a pathophysiological hallmark of VaD [1]. Moreover, recent neuroimaging studies have indicated significant hypoperfusion in the brain regions vulnerable to AD [9, 10]. Further experiments using normal and transgenic AD rodent models revealed that CCH increased the deposition of β-amyloid (Aβ) protein [11] and the level of phosphorylated tau protein [12], thus providing evidence for the vascular hypothesis of AD [13]. Among metabolic disorders, hyperlipidemia can jeopardize the function of small arteries and arterioles (arteriolosclerosis), and this could be exacerbated by hypertension and diabetes. The impaired autoregulation of these involved stiffened small cerebral vessels can ultimately lead to reduced cerebral blood flow and CCH [14]. Moreover, hyperlipidemia can trigger the formation of plaques in large and medium-size arteries (atherosclerosis) such as carotid arteries. Although a certain proportion of patients with carotid stenosis display no symptoms, they are at higher risks of mobility and cognitive dysfunction compared with those without stenosis, and this may often be attributed to CCH [15, 16]. Progressive brain injuries induced by CCH include metabolic disturbance [17], white matter injury, neuronal apoptosis and pyroptosis [18], synaptic plasticity impairment [19], activated neuroinflammation [20], and neuroendocrine perturbation [21]. Despite recent developments in experimental and clinical neuroscience, few options could be adopted to impede CCH-induced brain injury.

Bidirectional communications exist between the brain and the gut, with the recently found integration of gut microbiota [22]. Abundant commensal microbes inhabit the gastrointestinal lumen [23] and signal to the host’s immune and endocrine systems by secreting metabolites such as short-chain fatty acids (SCFAs), polyamines, and amino acids [24, 25]. In addition to regulating host homeostasis, gut microbiota plays a role in metabolic dysfunction, including obesity, diabetes mellitus, and cardiovascular diseases [26]. The microbiota-gut-brain axis is indispensable for normal central nervous system (CNS) development [27]. Specifically, microglial maturation depends on the continuous input of SCFAs from resident microbes, and depletion of the gut microbiome leads to microglial immaturity and malfunction [28]. SCFAs are small organic carboxylic acids with less than 7 carbon atoms and are mainly produced by the colonic bacterial fermentation of dietary fiber. Absorbed by intestinal epithelial cells, SCFAs participate in the host metabolism and are able to cross the blood-brain barriers via monocarboxylate transporters [29]. A variety of gut microbiota are responsible for SCFA fermentation [30]. It is reported that the depletion of acetate-producing bacteria including *Alistipes*, *Blautia*, *Ruminclostridium_9*, and *Roseburia* facilitated diabetes-induced hippocampal synaptophysin reduction [31], suggesting a role of gut microbiota in CNS injury triggered by metabolic disorders. There is accumulating evidence to indicate that CNS diseases and injuries can alter the composition of the gut microbiome [32–35]. Furthermore, transplantation of disrupted gut microbiome profiles from patients with schizophrenia and autism spectrum disorders into rodent animals could partially recapitulate related behaviors, disclosing the participation of gut microbiota dysbiosis in the progression of neurological disorders [36, 37]. Fecal microbiota transplantation (FMT) was first clinically applied for the treatment of pseudomembranous enterocolitis in 1958 [38]. Since then, an increasing number of clinical investigations have confirmed the positive therapeutic effects of FMT in gastrointestinal diseases, especially in inflammatory bowel diseases like ulcerative colitis and Crohn’s disease [39, 40]. As a result of new information regarding the microbiota-gut-brain axis, nervous and mental diseases have emerged as the second major FMT research area, although most of the current studies have been fundamental research.

The delicate gut homeostasis can easily be obliterated by acute stroke, either ischemic or hemorrhagic [24, 34, 41]. One of the indications is intestinal paralysis, possibly due to the catecholaminergic stress response [42, 43]. As a consequence of gut dysbiosis, altered gut microbiota composition might result in metabolite dysregulation, which can, in turn, aggravate brain injury [41]. And a positive effect has been proven through recolonization with more balanced microbiome [42] and interventions targeted at specific bacteria [34], including those that produce SCFAs [24]. Meanwhile, an investigation into the composition of fecal flora in 25 AD patients found that both the α and β diversities of gut microbiota in AD patients were significantly reduced compared with a control group matched in terms of age and gender [44]. Specifically, the levels of *Firmicutes* and *Actinomycetes* at the phylum level were decreased and *Bacteroidetes* was enriched in AD patients [44]. Aβ levels in germ-free Aβ precursor protein (APP) transgenic mice were lower than those in conventionally raised ones, which was reversed by colonization with microbiota from conventionally raised APP transgenic mice [45]. Another study demonstrated the beneficial effects of gut microbiota restoration on relieving cognitive impairment and associated pathological changes in an AD transgenic mouse [46]. The interaction of gut microbiome with post-stroke brain injury and AD has been unmasked. However, its correlation with CCH, the pathophysiological mechanism underlying VaD and AD to some extent, remains to be elucidated. Therefore, we aimed to explore whether brain injury caused by CCH could give rise to gut dysbiosis, and whether restoring gut microbial homeostasis could mitigate the effects of CCH. Investigations revealing the beneficial effect of *Clostridium butyricum* treatment in cerebral ischemia/reperfusion and permanent right unilateral common carotid arteries occlusion VaD mouse models also prompted our concentration at SCFA-producing floras [47–49]. In the present study, the classic bilateral common carotid artery occlusion (BCCAO) rat model was adopted to assess gut motility and barrier functions disruption and gut microbiota dysbiosis following relatively modest but prolonged brain injury caused by CCH. Our results provide further evidence that both modulation of the gut microbiome and long-term SCFA supplementation can improve CCH-induced intestinal malfunction, cognitive decline and depressive-like behaviors through anti-inflammation effects and the regulation of hippocampal apoptosis, thus shedding light on the neuroprotective potential of gut microbiome manipulation.

## Results

### BCCAO induces spatial learning and memory impairments and depressive-like behaviors

**Fig. 1:**
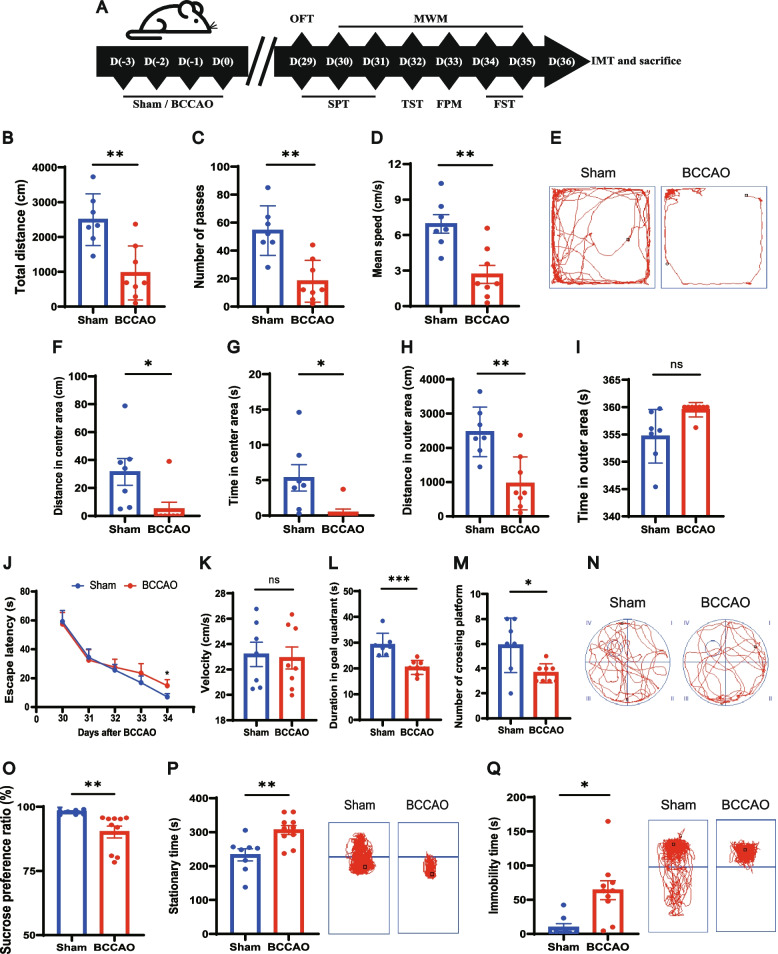
BCCAO induces spatial learning and memory impairments and depressive-like behaviors. **A** Timeline of the experiment (BCCAO, bilateral common carotid artery occlusion. OFT, open-field test. MWM, Morris water maze test. SPT, sucrose preference test. TST, tail suspension test. FPM, fecal paraments measurement. FST, forced swim test. IMT, intestinal motility test). **B**–**I** Open-field test (*n* = 7 Sham, *n* = 8 BCCAO): **B** The total distance traveled by rats in the open field. **C** The frequency of grid-crossing. **D** The mean traveling speed. **E** Representative traces in the open-field test. Circle dot: start position; square dot: end position. **F** Distance traveled in the center area. **G** Time spent in the center area. **H** Distance traveled in the outer area. **I** Time spent in the outer area. **J-N** Morris water maze test (*n* = 7 Sham, *n* = 8 BCCAO): **J** Escape latency during the 5-day training period. **K** Mean speed in water in the spatial probe test. **L** Time spent in and **M** frequency of visits to the quadrant where the platform had previously been located. **N** Representative traces in the spatial probe test (the platform was previously located in the center of quadrant IV). Circle dot: start position; square dot: end position. **O** Sucrose preference test (*n* = 8 Sham, *n* = 10 BCCAO): percentage of sucrose water intake. **P**, **Q** Duration of immobility and representative traces in the tail suspension test (**P**) and forced swim test (**Q**) (*n* = 8 Sham, *n* = 10 BCCAO). The data represent the mean ± SEM, *p* < 0.05 was set as the threshold for significance. * *p* < 0.05, ** *p* < 0.01, *** *p* < 0.001 compared to the sham group

Both sham and BCCAO rats were separated into two groups and underwent two batteries of behavioral tests (Fig. 1A). In the open-field test (OFT), which was conducted to investigate explorative and locomotor ability, the BCCAO rats displayed hypokinetic behaviors, with a decreased total traveled distance and number of passes, as well as a lower average speed of movement (Fig. 1B–E). Meanwhile, BCCAO rats spent less time and traveled shorter distance in center area (Fig. 1F–G). Although there was no significant difference between the two groups regarding the time spent in the outer area, the BCCAO rats traveled a shorter distance in the outer area, indicating a freezing state in depression (Fig. 1H, I). During the training period of the Morris water maze (MWM), a spatial learning difference was observed as the sham group presented with a shorter escape latency time, which reached statistical significance at day 34 post surgery (Fig. 1J). With the same swimming speed (Fig. 1K), less duration and fewer visits in the goal quadrant were noted in rats subjected to BCCAO, indicating spatial memory impairment (Fig. 1L–N).

Another battery of tests used for depressive-like behavior assessment were arranged reasonably in sequence to avoid disturbance (Fig. 1A). The results of the sucrose preference test (SPT) demonstrated a state of anhedonia in BCCAO rats (Fig. 1O). In the tail suspension test (TST) and forced swim test (FST), which create an inescapable circumstance of oppression, BCCAO rats exhibited a more conspicuous state of despair after unavailing struggling as reflected by their increased immobility time (Fig. 1P, Q).

### BCCAO triggers compromised gut function and microbial ecology

**Fig. 2:**
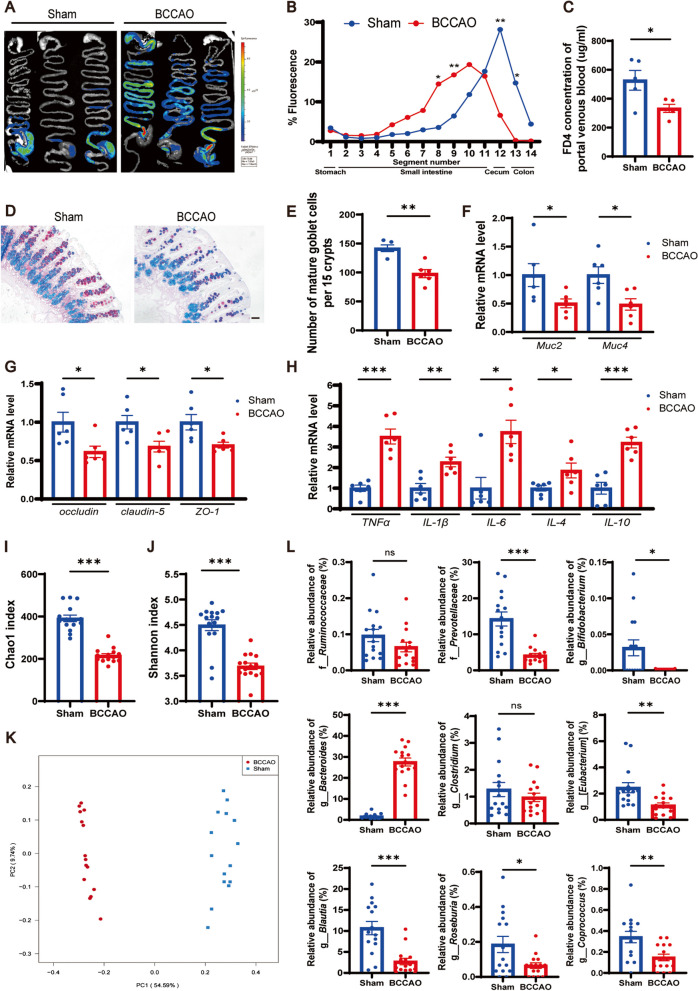
BCCAO disrupts gut function and microbial ecology. **A**–**C** Intestinal motility test (*n* = 5/group): **A** Representative images and **B** quantitative analysis showing the distribution of fluorescein isothiocyanate-dextran in gastrointestinal segments. **C** Concentration of fluorescein isothiocyanate-dextran in portal venous blood. **D** Alcian blue and periodic acid-Schiff (AB-PAS)-stained mature goblet cells from colon sections, and **E** calculation of mature goblet cells per 15 upper crypts/rat (*n* = 5 Sham, *n* = 6 BCCAO). Scale bar, 20 μm. **F-H** mRNA levels of mucins (*Muc2*, *Muc4*), tight junction proteins (*occludin*, *claudin-5*, and *ZO-1*), and inflammatory cytokines (*TNF-α*, *IL-1β*, *IL-6*, *IL-4*, and *IL-10*) in colon cells (*n* = 6/group). **I**, **J** Comparison of Chao1 index (**I**) and Shannon index (**J**) between the groups (*n* = 15/group). **K** Principal coordinate analysis (PCoA) plot established based on unweighted UniFrac distance (*n* = 15/group). **L** Bar plots of the relative abundance of f_*Ruminococcaceae* and f_*Prevotellaceae* and g_*Bifidobacterium*, g_*Bacteroides*, g_*Clostridium*, g_*[Eubacterium]*, g_*Blautia*, g_*Roseburia*, and g_*Coprococcus* (n = 15/group). The data represent the mean ± SEM, *p* < 0.05 was set as the threshold for significance. * *p* < 0.05, ** *p* < 0.01, *** *p* < 0.001 compared to the sham group

Body weight was measured before fecal parameters measurement (FPM) to exclude the possibility that BCCAO could exert an adverse effect on growth and development (Additional file 1: Fig. S1A). Among several fecal parameters, BCCAO rats had lower defecation frequency, fecal wet weight, fecal dry weight, and water content (Additional file 1: Fig. S1B-F), suggesting gastrointestinal dysfunction. Severe acute brain injury can lead to impaired gastrointestinal motility, which has been demonstrated on both clinical patients suffering from traumatic brain injury, acute ischemic stroke, and rodent models of acute middle cerebral artery occlusion, intracerebral hemorrhage [41, 42, 50, 51]. However, whether mild brain injury induced by CCH could influence intestinal motility remains unknown. Therefore, an intestinal motility test (IMT) was performed at day 36 post BCCAO prior to sacrifice and representative images are shown in Fig. 2A. Overall, fluorescein isothiocyanate-dextran (FD4) by gavage administration to BCCAO rats mainly remained in the lower intestinal segment while the sham group displayed higher fluorescence intensity in the cecum and colon segments (Fig. 2A, B). It was worth noting that one of the BCCAO rats was excluded from the analysis due to intestinal obstruction (Additional file 1: Fig. S2A). In addition, portal venous blood from BCCAO rats contained a lower level of FD4, which was a sign of reduced gut permeability (Fig. 2C).

Next, gut barrier function was assessed. Mature gut goblet cells produce mucins (Muc) to form a protective mucous layer in the lumen [52]. Histochemical staining using alcian blue and periodic acid-Schiff (AB-PAS) showed that the number of mature goblet cells in the colon was reduced in BCCAO rats (Fig. 2D, E). Muc2 and Muc4 respectively consist of inner and outer layers of mucus and their gene expression in large intestine mucosa was determined. Compared to the sham group, BCCAO rats had significantly lower expression of both the two genes (Fig. 2F). Next, mRNA levels of three tight junction proteins, namely *occludin*, *claudin-5*, and *zonula occludins-1* (*ZO-1*), were examined and the results showed their lower expression in BCCAO rats, suggesting a disrupted physical barrier of the large intestine (Fig. 2G). In addition, the intestinal immune state of BCCAO rats was perturbed as reflected by the upregulation of *tumor necrosis factor alpha* (*TNF-α*), *interleukin-1 beta* (*IL-1β*), *IL-6*, *IL-4*, and *IL-10* mRNA levels (Fig. 2H). Under normal physiological conditions, a balanced commensal microbiome helps repel foreign pathogens and also modulates intestinal endocrine and innate immune systems by inducing antimicrobial peptide production, acting as an intestinal microflora barrier [53]. Accordingly, the bacterial taxonomic composition was analyzed by 16S ribosomal RNA (rRNA) gene sequencing (original data provided in Additional file 2). The declined Chao1 index and Shannon index signified reduced microbial abundance and balance, respectively, in the BCCAO group (Fig. 2I, J). A conspicuous separation between the two groups was displayed in the unweighted UniFrac principal component analysis (PCoA) (Fig. 2K). As shown by the structure of gut microbiota at the phylum level (Additional file 1: Fig. S2B), the BCCAO rats possessed a higher relative abundance of *Bacteroidetes* and *Verrucomicrobia*, and lower levels of *Firmicutes* and *Tenericutes*. The linear discriminant analysis coupled with effect size (LEfSe) and heatmaps indicated significant differences in the microbial relative abundance at varied levels between the groups (Additional file 1: Fig. S3-5). And quantitative analysis revealed that some representative SCFA-producing bacteria were less enriched in the BCCAO rats, such as *Prevotellaceae* at the family level and *Bifidobacterium*, *Eubacterium*, *Blautia*, *Roseburia*, and *Coprococcus* at the genus level (Fig. 2L). These findings suggested that BCCAO could compromise gut function and cause microbial dysbiosis.

### BCCAO causes hippocampal injury, neuroinflammation, and SCFA reduction

**Fig. 3:**
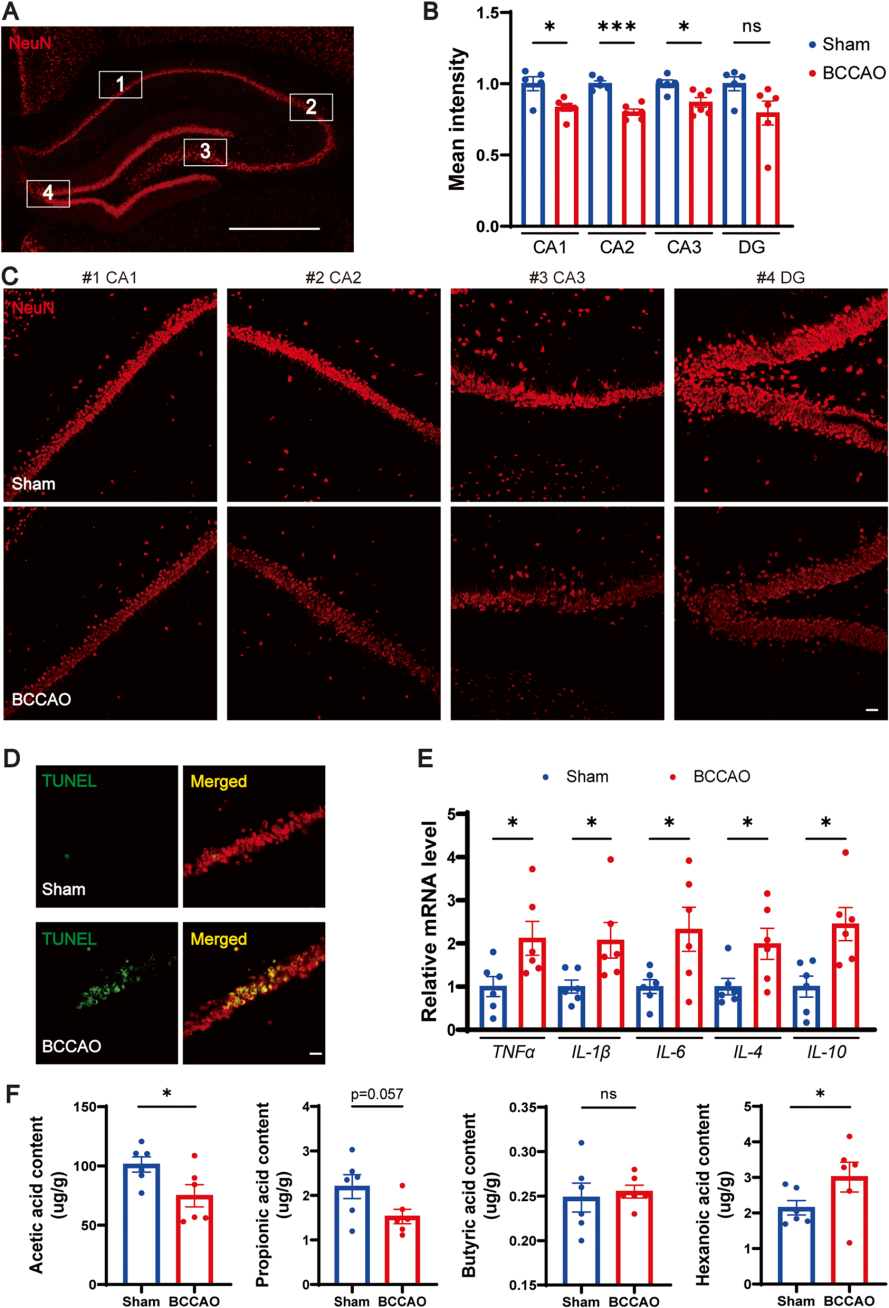
BCCAO causes hippocampal injury, neuroinflammation, and SCFA metabolic disorder. **A**–**C** Quantitative analysis of hippocampal neurons via immunofluorescence staining: **A** Immunofluorescence staining of hippocampal neuron. Scale bar, 1 mm. **B** Bar plots showing the mean fluorescence intensity (*n* = 5 Sham, *n* = 6 BCCAO) and **C** representative images of the cornu ammonis (CA) 1, CA2, CA3, and dentate gyrus (DG) zones. Scale bar, 50 μm. **D** TUNEL staining of hippocampal neurons. Scale bar, 30 μm. **E** mRNA levels of inflammatory cytokines (*TNF-α*, *IL-1β*, *IL-6*, *IL-4*, and *IL-10*) in the hippocampus (*n* = 6/group). **F** Bar plots showing hippocampal levels of acetic acid, propionic acid, butyric acid, and hexanoic acid (*n* = 6/group). The data represent the mean ± SEM, *p* < 0.05 was set as the threshold for significance. * *p* < 0.05, *** *p* < 0.001 compared to the sham group

Hippocampal changes underlie the cognitive decline in chronological aging and CNS diseases like AD [54, 55]. Previous studies have also correlated depressive disorders with abnormal hippocampus function [56–58]. Therefore, the hippocampal neuron was analyzed with immunofluorescence staining and the results showed that rats subjected to BCCAO showed weaker mean fluorescent intensity and decreased number of NeuN+ cells in the cornu ammonis (CA) 1, CA2, and CA3 regions, with a statistically insignificant tendency in the dentate gyrus (DG) (Fig. 3A–C, Additional file 1: Fig. S6A). TUNEL staining revealed that BCCAO increased the number of TUNEL-positive apoptosis hippocampal neurons (Fig. 3D). Consistent with the results of previous studies [18, 20], white matter injury after BCCAO was also revealed by the lower immunofluorescence intensity of myelin basic protein (MBP) on the corpus callosum, internal capsule, and caudoputamen (Additional file 1: Fig. S6B-D). In addition, a hippocampal neuroinflammatory response, characterized by significant overexpression of the five inflammatory cytokines, was noted in BCCAO rats (Fig. 3E). It was likely that microglia had passed through the activation period at day 36 post BCCAO as revealed by the relative downregulation of *ionized calcium-binding adaptor molecule-1* (*Iba-1*) and its inflammatory markers (Additional file 1: Fig. S6E). Since intestinally derived SCFAs must pass the blood-brain barrier through systemic circulation and take effect locally in the brain [59], hippocampal SCFA concentrations were straightly assessed using gas chromatography-mass spectrometry (GC-MS). Although several SCFAs (isobutyric acid, isovaleric acid, and valeric acid) were unmeasurable probably due to the insufficiency of sample mass for evaluation, a sign of SCFA metabolic disorder was found. To be specific, a downregulation of acetic acid, an upregulation of hexanoic acid, and a decreased (but not significant) level of propionic acid were determined in the hippocampus of BCCAO rats (Fig. 3F). These results showed the adverse influence of BCCAO on hippocampal neurons, neuroinflammation, and SCFA metabolism.

### FMT rebuilds differences in gut microbiota composition

**Fig. 4:**
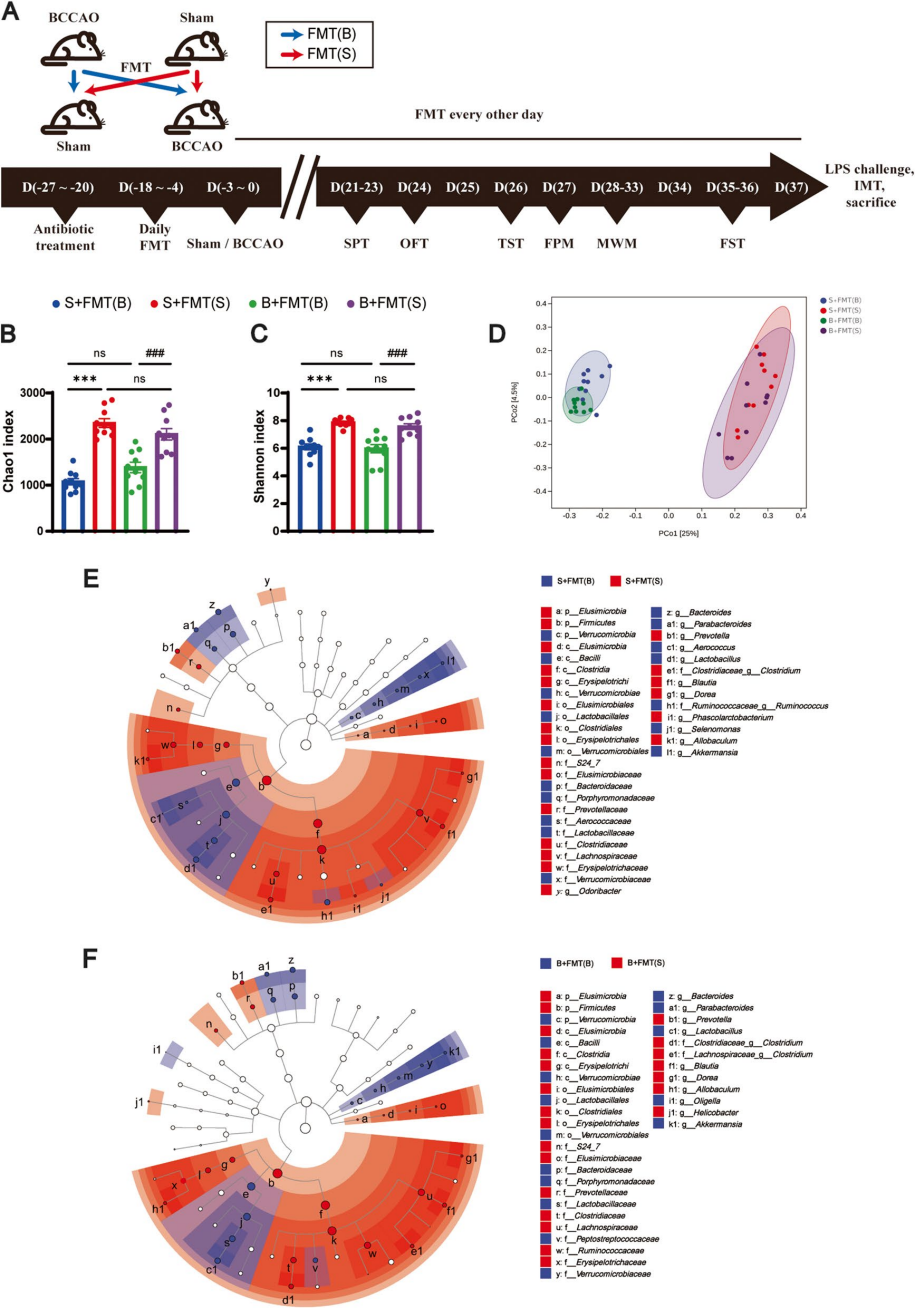
Fecal microbiota transplantation successfully establishes a varied gut microbiota profile. **A** Timeline of fecal microbiota transplantation (FMT) from BCCAO or sham animals to recipient rats (Blue arrows, FMT using fecal supernatant from BCCAO rats. Red arrows, healthy FMT using fecal supernatant from sham rats). **B**, **C** Comparison of the Chao1 index (**B**) and Shannon index (**C**) among the four groups (*n* = 10/group). **D** PCoA plot established based on the unweighted UniFrac distance (*n* = 10/group). **E**, **F** Representation of bacterial profiles in sham (**E**) and BCCAO (**F**) rats after receiving FMT by linear discriminant analysis coupled with effect size (*n* = 10/group). S+FMT(B), sham rats received BCCAO-rat-derived fecal microbiota transplantation. S+FMT(S), sham rats received sham-rat-derived fecal microbiota transplantation. B+FMT(B), BCCAO rats received BCCAO-rat-derived fecal microbiota transplantation. B+FMT(S), BCCAO rats received sham-rat-derived fecal microbiota transplantation. The data represent the mean ± SEM. *p* < 0.05 was set as the threshold for significance. *** *p* < 0.001 compared to the S+FMT(B) group. ^###^*p* < 0.001 compared to the B+FMT(B) group

To investigate the effects of perturbed and healthy gut microbiomes, FMT was performed on rats with or without BCCAO (Fig. 4A). In brief, 48 h after a 1-week antibiotic treatment in order to reduce the intestinal bacterial load, rats received a daily gavage of 1 mL gut microbiota supernatant for two weeks. The gut microbiota supernatant was prepared in advance with fresh feces from BCCAO or sham rats collected during behavioral tests, as depicted in Fig. 1A. The recipient rats were divided into two groups and subjected to sham surgery or BCCAO, respectively. Afterwards, FMT was conducted every other day until sacrifice in order to maintain the gut microbiota composition. Stool samples from the recipient rats were collected before sacrifice for 16S rRNA gene sequencing. The results showed that in both the sham and BCCAO groups, the rats that received fecal supernatant from sham rats had higher Chao1 and Shannon indexes compared with their counterparts (Fig. 4B, C). The unweighted UniFrac PCoA showed an obvious divergence between rats recolonized with balanced and imbalanced gut microbiota, with the subgroups overlapping to a great extent (Fig. 4D). The LEfSe analysis reflected the varied gut microbiota profile established between the recipient groups (Fig. 4E, F). A higher proportion of *Firmicutes*, *Proteobacteria*, *Tenericutes*, and *Spirochaetes* as well as a lower abundance of *Bacteroidetes*, *Verrucomicrobia*, and *Deferribacteres* at the phylum level were found in feces of the recipient rats with healthy fecal microbiota treatment (Additional file 1: Fig. S7A). Some of the differences in the compositions of SCFA-producers listed in Fig. 2L were also successfully established in at least one of the paired groups (Fig. 4E, F; Additional file 1: Fig. S7B), including the varied relative abundance of *Bacteroides*, *Bifidobacterium*, *Blautia*, and *Roseburia* at the genus level. Interestingly, an elevated relative abundance of f_*Ruminococcaceae* and g_*Clostridium* was identified in stool samples from BCCAO recipient rats with healthy FMT in comparison to those with perturbed FMT. But this phenomenon was absent in the donor fecal samples. Moreover, another two SCFA-producers, g_*Odoribacter* and g_*Dorea*, were undetected in the donor fecal samples probably due to an imperfect analytic method. However, their increased proportions were determined in rats after healthy FMT in both sham and BCCAO groups. The original data of 16S rRNA gene sequencing could be referred to in Additional file 3. The above results suggested that the difference in gut microbe composition was successfully rebuilt in the recipient rats through FMT.

### Restoration of gut microbiome alleviates cognitive decline and depressive-like behaviors post BCCAO

**Fig. 5:**
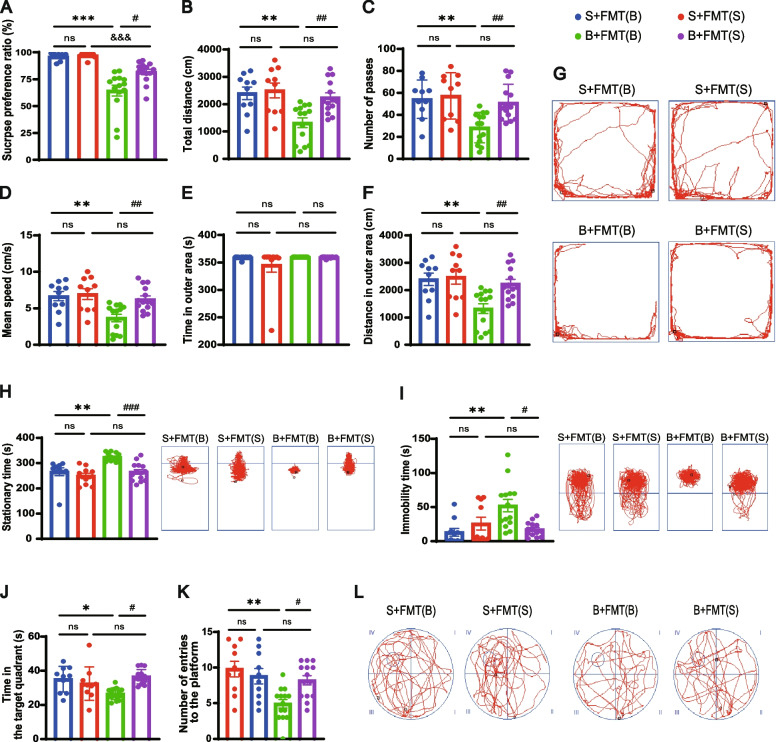
FMT alleviates depressive-like behaviors and cognitive decline post BCCAO. **A** Sucrose preference test. **B**–**G** Open-field test: **B** The total distance traveled by rats in the open-field test. **C** The frequency of grid-crossing. **D** The mean traveling speed. **E** Time spent in the outer area. **F** Distance traveled in the outer area. **G** Representative traces in the open-field test. Circle dot: start position; square dot: end position. **H**, **I** Duration of immobility and representative traces in the tail suspension test (**H**) and forced swim test (**I**). **J-L** Morris water maze test: **J** Time spent in and **K** frequency of visits to the quadrant where the platform was previously located. **L** Representative traces in the spatial probe test (the platform was previously located in the center of quadrant IV). Circle dot: start position; square dot: end position. The data represent the mean ± SEM (*n* = 10 S+FMT(B) and S+FMT(S), *n* = 14 B+FMT(B) and B+FMT(S)). *p* < 0.05 was set as the threshold for significance. * *p* < 0.05, ** *p* < 0.01, *** *p* < 0.001 compared to the S+FMT(B) group. ^&&&^*p* < 0.001 compared to the S+FMT(S) group. ^#^*p* < 0.05, ^##^*p* < 0.01, ^###^*p* < 0.001 compared to the B+FMT(B) group

Consistent with our previous results (Fig. 1O), both the BCCAO groups showed an obvious reduction in their preference for sucrose compared with their paired controls. However, this anhedonia was ameliorated by healthy FMT (Fig. 5A). The observed decrease in explorative and spontaneous activity in the BCCAO rats was improved by FMT, as revealed by the increase in total traveled distance, number of passes, and mean speed of BCCAO rats colonized with a balanced gut microbiome (Fig. 5B–D). An elevated traveled distance in the outer area observed in BCCAO rats with healthy FMT could indicate their alleviated freezing state (Fig. 5E–G). The anti-depressive effect of colonization with balanced gut microbiota was further confirmed by the TST and FST since it lowered the immobility time of BCCAO rats in both the two tests (Fig. 5H, I). In the MWM, no significant difference between the BCCAO groups was observed regarding escape latency. However, the BCCAO rats that received healthy FMT spent more time in, and visited more frequently, the target quadrant than those with perturbed FMT (Fig. 5J–L), reflecting their improved spatial memory defects.

### Healthy FMT improves gut motility and function in BCCAO rats

**Fig. 6:**
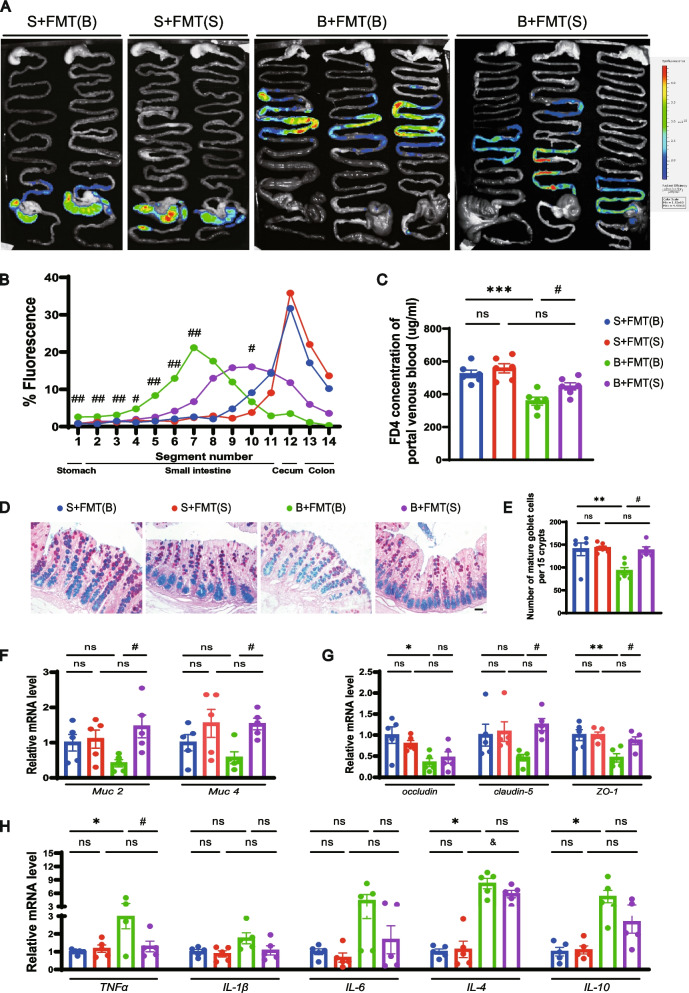
FMT improves gut motility and barrier functions in BCCAO rats. **A**–**C** Intestinal motility test (*n* = 6/group): **A** Representative images and **B** quantitative analysis showing the distribution of fluorescein isothiocyanate-dextran in gastrointestinal segments. **C** Concentration of fluorescein isothiocyanate-dextran in portal venous blood. **D** AB-PAS-stained mature goblet cells from colon sections, and **E** calculation of mature goblet cells per 15 upper crypts/rat (n = 5-6/group). Scale bar, 20 μm. **F**–**H** mRNA levels of mucins (*Muc2*, *Muc4*), tight junction proteins (*occludin*, *claudin-5*, and *ZO-1*), and inflammatory cytokines (*TNF-α*, *IL-1β*, *IL-6*, *IL-4*, and *IL-10*) in colon cells (*n* = 5/group). The data represent the mean ± SEM. *p* < 0.05 was set as the threshold for significance. * *p* < 0.05, ** *p* < 0.01, *** *p* < 0.001 compared to the S+FMT(B) group. ^&^*p* < 0.05 compared to the S+FMT(S) group. ^#^*p* < 0.05 compared to the B+FMT(B) group

Fecal parameters were measured to assess the overall gastrointestinal function after FMT. Recipient rats with or without BCCAO treatment had longer fecal pellets after healthy FMT (Additional file 1: Fig. S8C). In addition, colonization of balanced gut microbiota improved the decreased fecal wet weight and dry weight post BCCAO (Additional file 1: Fig. S8D, E). Next, to investigate the effect of FMT on gut motility, the IMT was conducted before sacrifice. Representative images from different groups were shown in Fig. 6A. Overall, the BCCAO rats displayed compromised gastrointestinal transit and permeability compared to the sham rats. By contrast, FMT with balanced microbiome enhanced gut peristalsis and permeability in BCCAO rats, indicated by weaker fluoresce intensity from the stomach to the upper intestine and stronger intensity in the lower intestine, and higher concentration of FD4 in the portal venous blood, respectively (Fig. 6A–C). AB-PAS histochemical staining of colon sections demonstrated that balanced microbiota transplantation increased the amount of mature goblet cells in BCCAO rats (Fig. 6D, E). Moreover, elevated mRNA levels of *Muc2*, *Muc4*, *claudin-5*, and *ZO-1* were found in the colons of healthy FMT-treated BCCAO rats, signifying more robust chemical and physical gut barriers (Fig. 6F, G). The downward trends in inflammatory cytokine expression, although only that of *TNF-α* reached a statistical significance, revealed a subsided inflammatory response post BCCAO by restoring gut flora (Fig. 6H).

### Treatment with balanced gut flora ameliorates BCCAO-induced hippocampal neuronal apoptosis and LPS-stimulated neuroinflammation

**Fig. 7:**
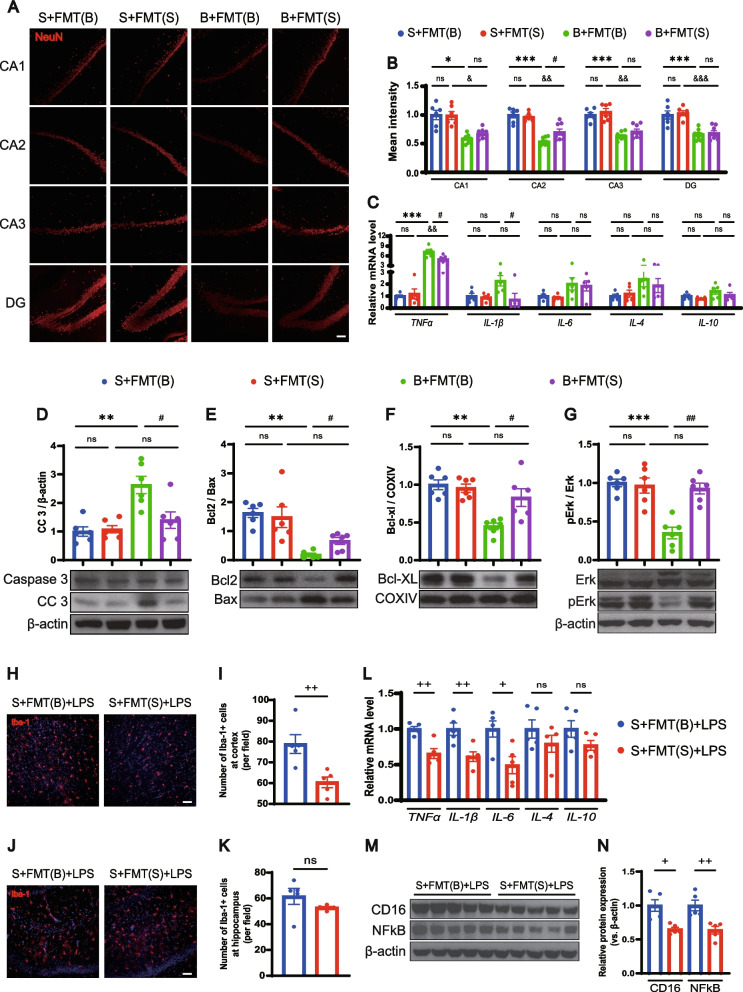
FMT ameliorates BCCAO-induced hippocampal neuronal apoptosis and LPS-stimulated neuroinflammation. **A** Representative immunofluorescence images of hippocampal neurons in CA1, CA2, CA3, and DG zones. Scale bar, 50 μm. **B** Bar plots of the mean fluorescence intensity in the different groups (*n* = 6 S+FMT(B) and S+FMT(S), *n* = 8 B+FMT(B) and B+FMT(S)). **C** mRNA levels of inflammatory cytokines (*TNF-α*, *IL-1β*, *IL-6*, *IL-4*, and *IL-10*) in the hippocampus (*n* = 5/group). **D**–**G** Western blot analysis for protein levels of caspase 3, cleaved caspase 3, Bcl2, Bax, Bcl-XL, Erk, and pErk and their quantification (band intensity normalized to β-actin or COXIV) in the hippocampus (*n* = 6/group). **H**–**K** Representative immunofluorescence images showing Iba-1+ cells in the cortex (**H**) and hippocampus (**J**), and the quantification analysis (**I**, **K**) (*n* = 5/group). Scale bar, 50 μm. **L** mRNA levels of inflammatory cytokines (*TNF-α*, *IL-1β*, *IL-6*, *IL-4*, and *IL-10*) in the cortex (*n* = 5/group). **M**, **N** Protein levels of CD16 and NF-κB and their quantification (band intensity normalized to β-actin) in the cortex (*n* = 5/group). S+FMT(B)+LPS, sham rats treated with LPS after receiving BCCAO-rat-derived fecal microbiota transplantation. S+FMT(S)+LPS, sham rats treated with LPS after receiving sham-rat-derived fecal microbiota transplantation. The data represent the mean ± SEM. *p* < 0.05 was set as the threshold for significance. * *p* < 0.05, ** *p* < 0.01, *** *p* < 0.001 compared to the S+FMT(B) group. ^&^*p* < 0.05, ^&&^ p < 0.01, ^&&&^ p < 0.001 compared to the S+FMT(S) group. ^#^ p < 0.05, ^##^*p* < 0.01 compared to the B+FMT(B) group. ^+^*p* < 0.05, ^++^*p* < 0.01 compared to the S+FMT(B)+LPS group

Hippocampal neuronal damage was reconfirmed by the decreased intensity and number of NeuN-positive cells in all CA and DG zones in the two BCCAO groups. Meanwhile, the balanced gut microbiota intervention enhanced the intensity and the number of NeuN-positive cells in hippocampal zones of the BCCAO group, especially in the CA2 area (Fig. 7A, B, Additional file 1: Fig. S9), indicating better neuronal survival. White matter injury could not be influenced by FMT (Additional file 1: Fig. S10). An eased inflammatory reaction in the hippocampus after healthy FMT was shown by reduced expression of *TNF-α* and *IL-1β* (Fig. 7C). In the canonical apoptotic pathway, proapoptotic molecules such as B cell lymphoma (Bcl) 2-associated X regulator (Bax) are activated, and the oligomerization of which triggers the leakage of cytochrome c from mitochondria into the cytoplasm [60]. Ultimately, caspase 3 can be cleaved into a functioning form that orchestrates both the cytoplasmic and nuclear alterations required for cellular disassembly, acting as a crucial executor [60, 61]. The results of western blotting confirmed remarkably elevated expression of cleaved caspase 3 (CC3) in the hippocampus post BCCAO, which was attenuated by the restoration of gut flora (Fig. 7D). Mitochondria were extracted from the hippocampus for analysis of pro-apoptotic and anti-apoptotic proteins. Healthy FMT significantly increased the Bcl2/Bax ratio and the expression of another anti-apoptotic protein-Bcl-XL (Fig. 7E, F).

Then hippocampal SCFA levels were determined. Specifically, recipient rats with balanced FMT had a significantly higher hippocampal acetic acid content, be it in the sham or BCCAO group. Moreover, similar trends of propionic acid, butyric acid, isovaleric acid, and valeric acid were observed in at least one of the paired groups (Additional file 1: Fig. S11). Therefore, FMT comprising a relatively higher abundance of SCFA producers improved SCFA fermentation to some extent.

SCFAs are ligands of G protein-coupled receptors (GPRs) [62–64], one of which, GPR41, was found to express in cortical neurons of rats [65]. In this study, colocalization of GPR41 and hippocampal neurons was revealed by immunofluorescence staining (Additional file 1: Fig. S12A). And the balanced gut microbiota intervention reversed the dephosphorylation of p44/42 mitogen-activated protein kinase (Erk1/2) in the hippocampus following BCCAO, indicating a GPR41 mediated activation of the Erk1/2 cascade (Fig. 7G), which is consistent with previous reports [64, 66]. The above data demonstrated that SCFA-producing flora enrichment through FMT could ameliorate BCCAO-induced hippocampal neuronal apoptosis via Erk1/2 activation.

The immunomodulatory and anti-inflammation effects of SCFAs have been previously reported [24, 67]. Compromised microbiota complexity can lead to defective microglia and constant SCFA input is indispensable for microglia homeostasis [68]. Moreover, the expression of GPR41 on microglia was identified by immunofluorescence staining (Additional file 1: Fig. S12B). We proposed that balanced gut flora colonization with more SCFA-producers could regulate the classically lipopolysaccharide (LPS)-stimulated microglia activation. A batch of rats from sham groups with FMT treatment were subjected to LPS intraperitoneal injection and, to our surprise, immunofluorescence staining suggested a significantly smaller number of Iba-1+ microglia at the cortex, but not the hippocampus, under healthy FMT (Fig. 7H–K). The mRNA expression of *TNF-α*, *IL-1β*, and *IL-6* and the protein expression of pro-inflammatory microglia marker Fc RII/III receptor (CD16) were relatively down-regulated in the cortex of rats colonized with more SCFA-producers (Fig. 7L–N). Meanwhile, a substantial decrease in cortical nuclear factor kappa beta (NF-κB) protein expression was found in sham rats with healthy FMT (Fig. 7M, N). Therefore, healthy FMT could regulate microglia activation and exert an anti-inflammation effect through NF-κB inhibition.

### SCFA supplementation improves spatial memory and depressive-like behaviors after BCCAO

**Fig. 8:**
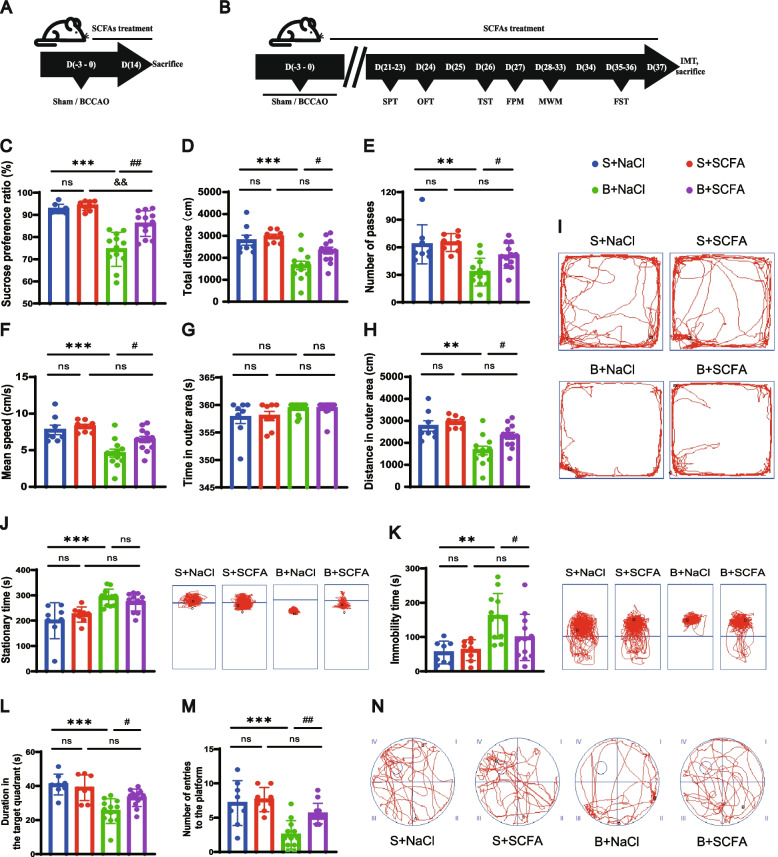
Long-term SCFA supplementation improves depressive-like behaviors and spatial memory after BCCAO. **A**, **B** Timeline of the experiment. **C** Sucrose preference test. **D**–**I** Open-field test: **D** The total distance traveled by rats in the open field. **E** The frequency of grid-crossing. **F** The mean traveling speed. **G** Time spent in the outer area. **H** Distance traveled in the outer area. **I** Representative traces in the open-field test (Circle dot: start position; square dot: end position). **J**, **K** Duration of immobility and representative traces in the tail suspension test (**J**) and forced swim test (**K**). **L**–**N** Morris water maze test: **L** Time spent in and **M** frequency of entries into the quadrant where the platform was previously located. **N** Representative traces in the spatial probe test (the platform was previously located in the center of quadrant IV). Circle dot: start position; square dot: end position). S+NaCl, sham rats treated with sodium chloride (NaCl). S+SCFA, sham rats that received short-chain fatty acids (SCFAs) (acetate, propionate, and butyrate) supplementation. B+NaCl, BCCAO rats treated with NaCl. B+SCFA, BCCAO rats received SCFAs supplementation. The data represent the mean ± SEM (*n* = 8 S+NaCl and S+SCFA, *n* = 12 B+NaCl and B+SCFA). *p* < 0.05 was set as the threshold for significance. ** *p* < 0.01, *** *p* < 0.001 compared to the S+NaCl group. ^&&^*p* < 0.01 compared to the S+SCFA group. ^#^*p* < 0.05, ^##^*p* < 0.01 compared to the B+NaCl group

Given the above results, we hypothesized that SCFAs could mediate the beneficial influence of gut flora recolonization. To test this hypothesis, rats were supplemented consecutively with either drinking water containing a mix of three main components of SCFAs (acetate, propionate, and butyrate) or control drinking water with a matched sodium chloride concentration, for short (Fig. 8A) and long periods (Fig. 8B). After long-term SCFA treatment, BCCAO rats displayed a higher sucrose preference ratio than the control group (Fig. 8C). In addition, SCFA intake led to an increase in the total traveled distance, grid crossing, and mean velocity of BCCAO rats in the OFT (Fig. 8D–F). Their locomotor in the outer zone was improved as well (Fig. 8G–I). The descended immobility time in the FST, though without that in the TST, further verified the effects of SCFAs on relieving depressive-like behaviors (Fig. 8J, K). Furthermore, both the declined time spent in and the reduced entries into the target quadrant of BCCAO rats were reversed by the SCFA intervention (Fig. 8L–N). Thus, the behavioral data disclosed the beneficial effect of SCFAs on depression and cognitive recovery post BCCAO.

### SCFA supplementation counteracts disruptions of gut motility and function

**Fig. 9:**
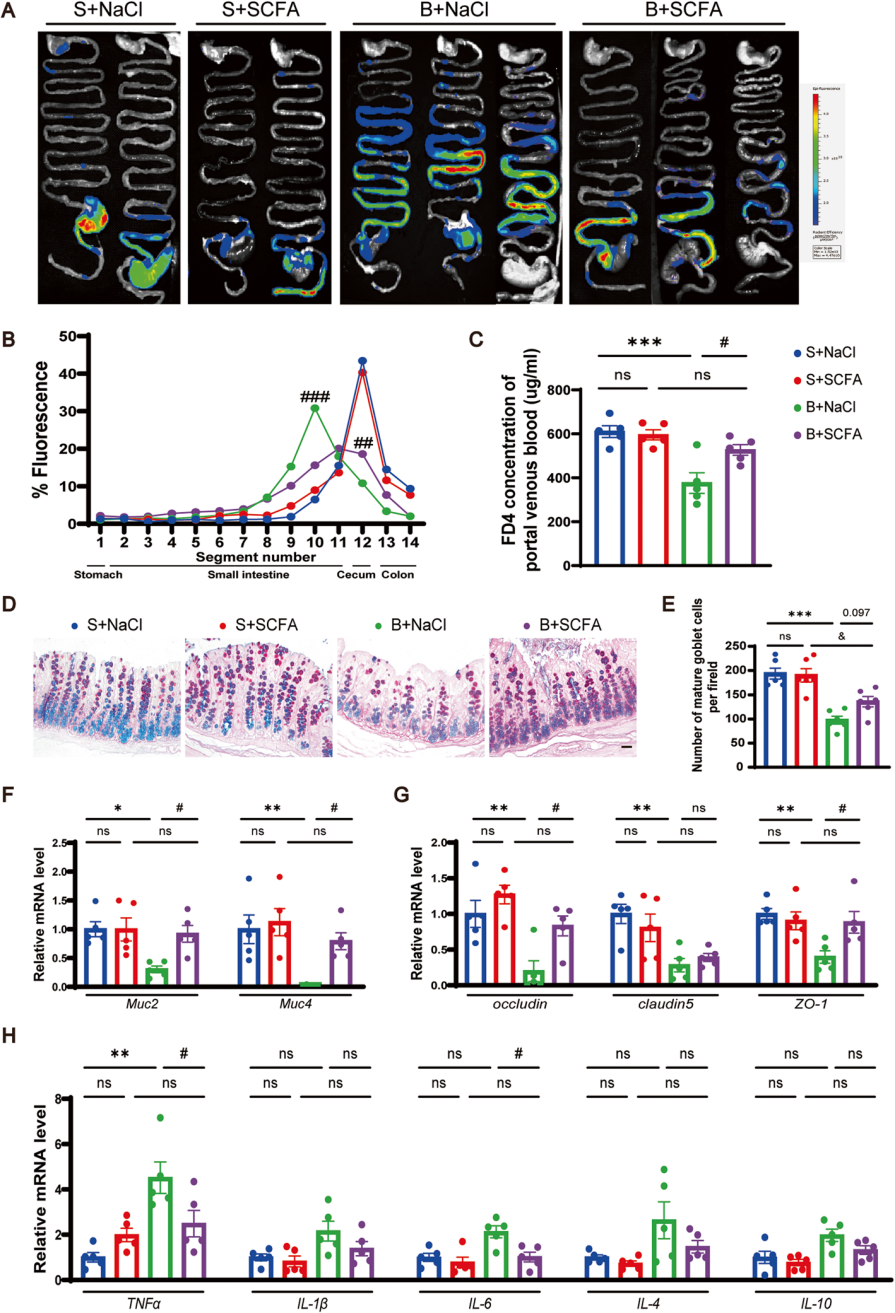
Long-term SCFA supplementation ameliorates compromised gut motility and barrier functions in BCCAO rats. **A**–**C** Intestinal motility test (*n* = 5/group): **A** Representative images and **B** quantitative analysis showing the distribution of fluorescein isothiocyanate-dextran in gastrointestinal segments. **C** Concentration of fluorescein isothiocyanate-dextran in portal venous blood. **D** AB-PAS-stained mature goblet cells from colon sections, and **E** calculation of mature goblet cells per 15 upper crypts/rat (*n* = 6/group). Scale bar, 20 μm. **F-H** mRNA levels of mucins (*Muc2*, *Muc4*), tight junction proteins (*occludin*, *claudin-5*, and *ZO-1*), and inflammatory cytokines (*TNF-α*, *IL-1β*, *IL-6*, *IL-4*, and *IL-10*) in colon cells (*n* = 5/group). The data represent the mean ± SEM. *p* < 0.05 was set as the threshold for significance. * *p* < 0.05, ** *p* < 0.01, *** *p* < 0.001 compared to the S+NaCl group. ^&^*p* < 0.05 compared to the S+SCFA group. ^#^*p* < 0.05 compared to the B+NaCl group

SCFA supplementation did not affect body weight or fecal parameters (Additional file 1: Fig. S13). In the gastrointestinal transit assessment, the BCCAO rats with SCFA treatment displayed less retention of FD4 in the small intestine (Fig. 9A, B), as well as a higher concentration of FD4 in the portal venous blood, compared with the control BCCAO group (Fig. 9C). The decrease of mature colon goblet cells shown by AB-PAS histochemical staining in the BCCAO rats was ameliorated by SCFAs to some extent, without reaching a statistical significance (Fig. 9D, E). Furthermore, SCFA intake prompted the expression of *Muc2*, *Muc4*, *occludin*, and *ZO-1*, while lowering that of *TNF-α* and *IL-6* in the large intestine mucosa in BCCAO rats (Fig. 9F–H). Altogether, SCFA supplementation could promote gastrointestinal transit and modulate gut barrier function after BCCAO.

### SCFA supplementation mitigates neuroinflammation and promotes neuronal survival in hippocampus post BCCAO

**Fig. 10:**
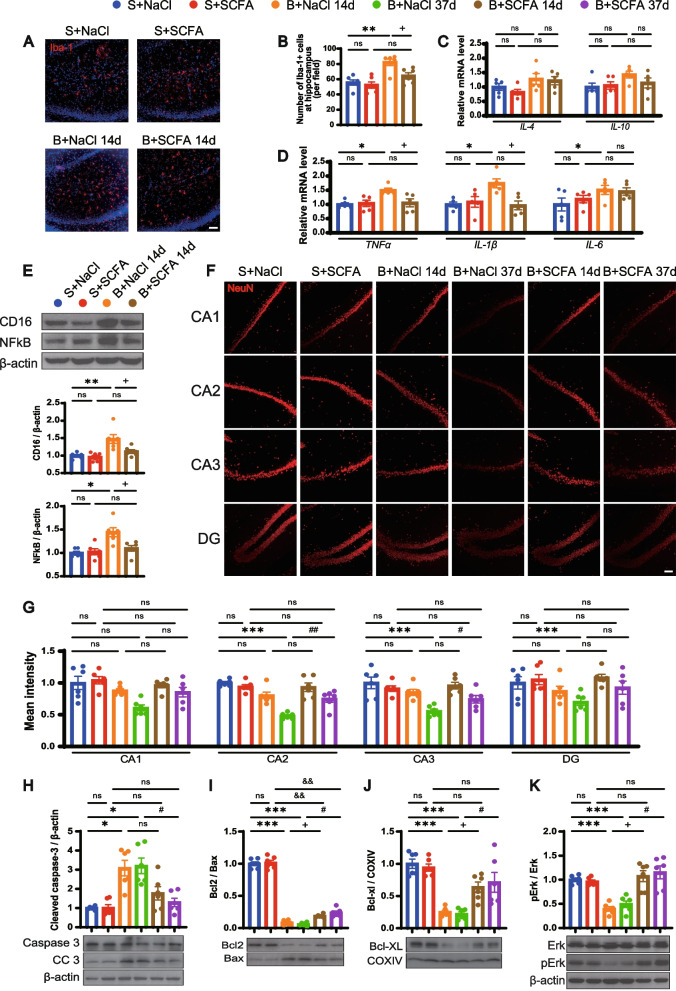
SCFA supplementation mitigates neuroinflammation and promotes neuronal survival in the hippocampus post BCCAO. **A**, **B** Representative immunofluorescence images showing Iba-1+ cells in the hippocampus at day 14 post BCCAO (**A**) and the quantification analysis (**B**) (*n* = 6/group). Scale bar, 50 μm. **C**, **D** mRNA levels of inflammatory cytokines (*IL-4*, *IL-10, TNF-α*, *IL-1β*, and *IL-6*) in the hippocampus at day 14 post BCCAO (*n* = 5/group). **E** Protein levels of CD16 and NF-κB and their quantification in the hippocampus at day 14 post BCCAO (band intensity normalized to β-actin) (*n* = 6/group). **F**, **G** Representative immunofluorescence images (**F**) and mean fluorescence intensity (**G**) of hippocampal neurons in CA1, CA2, CA3, and DG in the different groups. (*n* = 6/group). Scale bar, 50 μm. **H**–**K** Western blot analysis for protein levels of caspase 3, cleaved caspase 3, Bcl2, Bax, Bcl-XL, Erk, and pErk and their quantification (band intensity normalized to β-actin or COXIV) in the hippocampus (*n* = 6/group). The data represent the mean ± SEM. *p* < 0.05 was set as the threshold for significance. * *p* < 0.05, ** *p* < 0.01, *** *p* < 0.001 compared to S+NaCl group. ^&&^*p* < 0.01 compared to the S+SCFA group. ^+^*p* < 0.05 compared to the B+NaCl 14d group. ^#^*p* < 0.05, ^##^*p* < 0.01 compared to the B+NaCl 37d group

Resident immune cell-mediated neuroinflammation in the relatively early period post BCCAO was previously revealed by the upregulation of microglial pro-inflammatory markers [20]. As our previous results implied that SCFAs could contribute to microglia homeostasis after LPS stimulation (Fig. 7H–N), we hypothesized that BCCAO-induced microglia-mediated neuroinflammation would be abated by SCFA supplementation. After consecutive treatment with sodium chloride or SCFAs for 14 days (Fig. 8A), sham and BCCAO rats were sacrificed and immunofluorescence staining signified an elevated amount of Iba-1-positive cells in the hippocampus post BCCAO (Fig. 10A, B). However, this was alleviated by SCFA supplementation (Fig. 10A, B). A substantial downregulation of *TNF-α*, *IL-1β*, and *CD16* was observed in the hippocampus of BCCAO rats with SCFA treatment, compared with the control BCCAO group (Fig. 10C–E). Moreover, SCFAs significantly inhibited the BCCAO-induced upregulation of NF-κB (Fig. 10E), which is consistent with the results of the FMT intervention.

After that, the mean fluorescence intensity and the number of hippocampal NeuN+ cells were quantified. Overall, lower mean fluorescence intensity and decreased number of hippocampal NeuN+ cells were observed starting from 14 days post BCCAO and reached statistical significance at CA2, CA3, and DG regions at 37 days (Fig. 10F, G, Additional file 1: Fig. S14). But these downward trends were impeded by SCFA treatment, indicating better hippocampal neuron survival (Fig. 10F, G, Additional file 1: Fig. S14). Similarly, lower hippocampal protein expression of CC3 was noted in SCFA-supplemented BCCAO rats, compared with those of sodium chloride-treated BCCAO groups (Fig. 10H). The elevated Bcl2/Bax ratio and boosted expression of Bcl-XL also demonstrated a regulating effect of SCFAs on hippocampal neuron apoptosis (Fig. 10I, J). Furthermore, activation of Erk1/2 was confirmed to have begun by 14 days post BCCAO with SCFA supplementation and was ongoing at 37 days (Fig. 10K). The above data indicates that SCFAs suppressed the NF-κB pathway and activated an Erk1/2 cascade, which subsequently mitigated neuroinflammation and neuronal apoptosis, respectively, in the hippocampus following injury.

## Discussion

In the present study, in addition to cognitive impairment and depressive-like behaviors, we found that BCCAO led to compromised gut barrier function and perturbed gut microbiota in rats. We found reductions in representative SCFA-producing flora and hippocampal SCFAs in BCCAO-treated rats. After we conducted FMT, 16S rRNA gene sequencing confirmed the successful reproduction of microbial compositions in combined antibiotics-pretreated recipient rats, in both the sham and BCCAO groups. The transplantation of the balanced gut microbiome from sham rats not only improved disrupted gastrointestinal motility and gut dysbiosis but also ameliorated abnormal behaviors and hippocampal neuron apoptosis in BCCAO rats, probably via the anti-apoptotic effect of SCFAs. Furthermore, sham rats recolonized with perturbed gut microbes displayed more severe neuroinflammation than those with balanced gut microbiomes when subjected to an intraperitoneal injection of LPS, implying that SCFAs have anti-inflammation properties. Further experiments with SCFA supplementation demonstrated their beneficial effects on gut dysbiosis, cognitive decline, and depressive-like behaviors following BCCAO. Our data suggest that SCFA intake alleviated BCCAO-induced hippocampal inflammation and neuronal apoptosis via GPR41-mediated NF-κB inhibition and Erk1/2 activation, respectively.

Instead of referring to a specific cerebral disease, CCH actually underlies the secondary brain injury associated with a variety of CNS diseases and metabolic disorders. For example, Moyamoya disease (MMD) is a chronic cerebrovascular disease characterized by progressive stenosis of the arteries of the circle of Willis, with the formation of a collateral vascular network at the base of the brain [69]. Patients with MMD may present with either cerebral hemorrhage due to the fragile nature of the collateral vascular network, or ischemic stroke because of the irreversible and progressive arterial stenosis (or even occlusion). Without definite etiologies, the current treatment depends on cerebrovascular reconstructive surgery via a direct and/or indirect bypass approach [70]. However, MMD is, essentially, a chronic ischemic cerebrovascular disease. The exploration from the perspective of CCH could help unmask its pathogenesis and find potential therapeutic options. Consisting of the two leading forms of senile dementia, AD and VaD often coexist with each other, confirmed by autopsy studies and termed as mixed dementia [71]. Accumulating investigations have revealed that one of their shared pathological mechanisms is CCH [9, 10, 72]. CCH incurs negative impacts like oxidative stress, neuroinflammation, mitochondrial dysfunction, and metabolic dysfunction, aggravating brain injury insidiously [73, 74]. However, unlike acute ischemic stroke, it is likely that the relatively modest but prolonged nature of CCH could offer a time window for interventions to alleviate secondary damage.

**Fig. 11:**
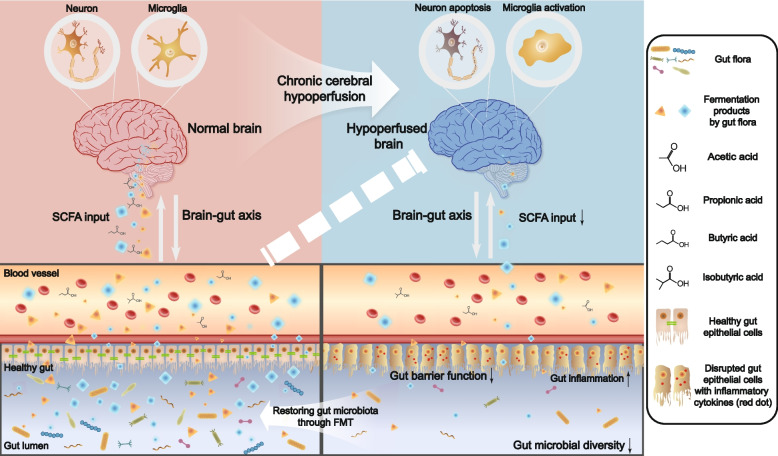
A schematic diagram for this study. Bidirectional communications exist between the brain and the gut, with the involvement of gut microbiota through fermented metabolites such as short-chain fatty acids (SCFAs). Brain injury caused by chronic hypoperfusion induces gut dysbiosis, accompanied with decreased fermentation products. Restoring gut microbiota through fecal microbiota transplantation (FMT) alleviates disrupted gut function, neuronal apoptosis, and neuroinflammation

Different floras compete for survival, and this must be taken into consideration when trying to reshape their composition. Treatment with one or several specific bacteria would probably lead to a relatively low survival rate. The strictly anaerobic conditions necessary for culturing a single bacterium could also boost experimental difficulty. To avoid these obstacles, we performed recolonization of whole fecal microbiota in the present study. And the recipient rats were pretreated with a mixture of unabsorbable antibiotics in order to reduce bacterial load, which, however, would pose a bias. To exclude the possible bias associated with gut flora depletion and the FMT process itself, rats in control groups were also transplanted with the corresponding gut microbiota. Furthermore, the recipient rats were treated with FMT every other day until sacrifice to sustain the rebuilt gut microbiota composition. Considering that CCH might not directly represent a specific disease, we chose the classic BCCAO rats to model CCH, aiming to explore from the pure perspective of chronic cerebral ischemia. And FMT was performed using stool samples from the rats instead of patients with uncharacteristic diseases for exclusion of confounding factors. Moreover, we speculated that the survival rate of the transplanted bacteria would be higher in animals of the same species. This was supported by our finding that FMT not only led to rebuilt bacterial compositions in sham rats but also induced reversals of the alterations caused by BCCAO. In our study, the recipient rats recolonized with the perturbed gut microbiota did not show obvious abnormal behaviors or any changes in gut functions except for a shorter fecal length. However, these rats displayed more serious inflammatory reactions following exposure to LPS. And sustaining a balanced gut microbiome composition via frequent FMT could improve cognitive ability, depressive-like behaviors, and gut functions after BCCAO. These data imply that gut microbiota dysbiosis could serve as an accomplice or contributor, rather than an initiator, in BCCAO-induced CCH. Thus, restoring gut microbiota may not eradicate a disease itself, but could reduce secondary damage caused by impaired signaling from the gut to the brain (Fig. 11).

In our study, 16S rRNA gene sequencing identified some of the representative SCFA-producing bacteria that were less enriched in the fecal samples from BCCAO rats. Among them, the g_*Blautia* and most groups of f_*Prevotellaceae* are acetic acid-derived bacteria while g_*Roseburia* and g_*Coprococcus* are both butyric acid-derived. Some groups of g_ [*Eubacterium]* take part in butyric acid fermentation. And g_*Bifidobacterium* could produce the three main components of SCFAs: acetic, propionic, and butyric acids. Most of these bacteria with different relative abundance were successfully transferred to recipient rats by FMT. Furthermore, rats that underwent FMT treatment acquired a varied tendency of hippocampal SCFA levels both in sham groups and BCCAO groups, although only the difference in the level of acetic acid reached a statistical significance. Previous studies have reported the beneficial effects of SCFAs on regulating microglia maturation and relieving post-stroke brain injury through attenuating neuroinflammation and neuronal apoptosis [24, 28, 65, 75]. Our data imply that a balanced gut microbiota recolonization can exert a protective effect against neuroinflammation by LPS exposure and hippocampal neuronal apoptosis caused by BCCAO, probably due to increased hippocampal SCFA levels. Meanwhile, the expression of one SCFA receptor, GPR41, was identified in both microglia and hippocampal neurons. We further verified the role of SCFAs by supplementing rats consecutively with drinking water containing a mix of the three main components of SCFAs by referring to another investigation [75]. And SCFA intake alleviated BCCAO-induced hippocampal neuroinflammation and neuronal apoptosis by suppressing NF-κB and activating Erk1/2, accompanied by improved cognitive ability and depressive-like behaviors.

One limitation of this intervention is that the intake of SCFAs for each rat could not be normalized. Nevertheless, SCFA supplementation in drinking water is consistent with the physiological process that the majority of SCFAs is fermented and absorbed from the intestine originally. Moreover, it prevents animals from stress and pain, compared with other methods of administration. Due to the short half-life of the saline molecule used [76, 77], this strategy of SCFA supplementation requires a high concentration in drinking water, which, however, boosts the intake of sodium. By contrast, restoring gut microbiota by FMT is a more reasonable approach to sustain the continuous production and input of these beneficial metabolites. On the other hand, to exclude the possible subsequent bias and rebuild the targeted gut microbiome profile, the time-consuming antibiotics pretreatment and pre-FMT were conducted prior to surgery. And considering the unknown long-term impact of BCCAO, frequent FMT was performed post BCCAO to sustain the established gut microbiota profile. Therefore, it is difficult to tell whether FMT exerts a protective effect or therapeutic effect on CCH, or probably both, which is another limitation of our experimental design. This could be resolved by the development of the FMT technique and a more reasonable experimental scheme in the future. Another limitation of this study is that we only used male animals. However, studies have revealed that exclusion of female rodents from studies simply due to estrous cycle variability may bring investigation bias and limit translational application of the findings [78, 79]. Moreover, in fact, stroke and some of the central nervous system diseases with chronic cerebral hypoperfusion as the pathophysiological hallmark mainly occur in the elderly. Our next research will be conducted using aged mice/rats, especially the reproductively senescent female ones.

## Conclusion

The current study indicated that BCCAO rats displayed compromised gut motility, disrupted gut barrier function, and perturbed gut microbiota, accompanied with cognitive impairment and depressive-like behaviors. Modulating gut microbiota by FMT with an increasing SCFA level as the underlying mechanism not only alleviated neuroinflammation by LPS exposure but also ameliorated cognitive decline and depressive-like behaviors following BCCAO by inhibiting hippocampal neuronal apoptosis, which was confirmed by further experiment using direct SCFA intervention.

## Materials and methods

### Animal model

All experiments described in this study were approved by the Animal Care and Use Committee of Fudan University (China) and carried out according to the Regulations for Laboratory Animal Management by the Ministry of Science and Technology of the People’s Republic of China. Adult male Sprague-Dawley rats (3 months old, 250–300 g) were purchased from JSJ Laboratory Animals, Shanghai, China and housed in groups of 4–6 in environmentally controlled conditions (a room temperature of 20–22 °C, 50% humidity, and under a 12-h light/dark cycle), with free access to water and food.

CCH was induced by BCCAO in accordance with previous studies [20, 80] with slight modifications to reduce mortality. Briefly, the rats were initially anesthetized with 5% isoflurane in 70% nitrogen and 30% oxygen and maintained with 2% isoflurane in 0.5 L/min oxygen. Through a midline incision, the right common carotid artery (CCA) was exposed and carefully separated from the sheath and vagus nerve, followed by double ligation with 5-0 silk sutures and cutting between the ligations in case of recanalization. The same surgical procedure was conducted on the left CCA 72 h later through the same incision. The sham group underwent the same surgical procedures without occlusion of the carotid arteries. During the entire surgical procedure, the rectal temperature was maintained at 36.5–37.5 °C using a heat pad. After surgery, the rats recovered from anesthesia and were returned to their cages.

### Behavioral testing

Behavioral testing was performed between 9:00 AM and 17:00 PM. Before testing, all rats were placed in the testing room, while still in their home cages, for 30 min of habituation to minimize the effects of novelty or stress. The facilities were cleaned with 75% ethanol between trials. The results were analyzed using the Xmaze video tracking system (Shanghai XinRuan Information Technology Co., Ltd., China) or scored manually.

#### Open-field test

The rats were placed in an open-field arena (100 × 100 × 45 cm) and allowed to explore freely for 6 min. The open area was divided into 16 square fields, and the 4 middle square fields were denoted as the central area. The distance traveled and the time spent in and out of the central zone were calculated.

#### Morris water maze test

A black circular pool (150 cm in diameter, 50 cm in height) filled with water at 23 ± 1 °C to a depth of 45 cm was divided into four equal quadrants by four equidistant points on the wall. A transparent escape platform was submerged approximately 1.5–2.0 cm below the water surface and located in the center of quadrant IV. Visual cues fixed at set positions could be used for spatial orientation.

In the acquisition trial phase, which took place during the first consecutive five days, the rats were gently placed in one of the four designated start positions, facing the wall of the maze, and the amount of time required to find the hidden platform (the escape latency) was recorded. A rat would be guided to the platform with an additional stay for 10 s and a maximum score of 120 s was assigned when it failed to locate the platform. Four trials were conducted per day, and each one had a maximum time of 2 min with an interval of 15 s. On day 6, the probe trial was conducted by placing each rat in a new start position to swim in the pool without the platform for 120 s. The time spent in and the number of entries into the target quadrant where the platform was previously located were recorded.

#### Sucrose preference test

Individually housed rats were provided with 1% sucrose solution in two bottles. Twenty-four hours later, one of the bottles was replaced with plain water. The positions of the two bottles were swapped every 4 h in case of location preference. Then, following 12 h of food and water deprivation, the rats were given free access to two bottles, one containing 1% sucrose solution and one containing plain water, for 12 hours. The bottles were weighed, and the preference of sucrose was calculated using the following formula: sucrose consumption (g) / consumption of [sucrose (g) + water (g)] × 100%.

#### Tail suspension test

The rats were suspended from a shelf (70 cm tall, 30 cm wide) 20–25 cm above the ground for 6 min. They were attached via adhesive tape placed 2–3 cm from the tip of the tail. The duration of climbing and struggling, as well as the time spent immobile, were recorded.

#### Forced swim test

On the first day (training day), the rats were individually placed in an open, clear Plexiglas cylindrical container (50 cm tall, 30 cm wide) containing 35 cm of water at 25±1 °C, and forced to swim for 15 min. Then they were removed and dried before being returned to their home cages. Twenty-four hours later (test day), the rats were forced to swim for 6 min, and the duration of active behaviors (climbing and swimming) and immobility (floating) were recorded.

### Fecal collection and 16S rRNA gene sequencing analysis

Fresh stool samples from rats were collected in sterile tubes and immediately frozen in liquid nitrogen, and then stored at − 80 °C until analysis. The bacteria taxa in each stool sample were analyzed by amplifying the V4 to V5 regions of the 16S rRNA gene using Quantitative Insights Into Microbial Ecology (QIIME, v1.9.0, http://qiime.org/) and the R package 3.5.1 (https://www.r-project.org/). Being clustered into operational taxonomic units (OTUs) with 97% sequence similarity or amplicon sequence variants (ASVs) with 100% sequence similarity, reads of high quality were selected for bioinformatics analysis. Alpha diversity was calculated based on the Chao1 and Shannon indexes. Beta diversity analysis was performed to assess the structural variations of microbial communities between the experimental groups using UniFrac distance metrics and visualized via principal coordinate analysis. Linear discriminant analysis and heatmaps were performed to detect differentially abundant taxa at varied levels across the groups.

### Fecal microbiota transplantation

#### Donor rats and harvesting of feces

Feces from donor rats were collected and pooled, followed by dilution with chilled phosphate-buffered saline solution (PBS; 100 mg feces/1 mL buffer). Then the samples were homogenized for 10 min to achieve a paste-like consistency, vortexed for 1 min, and centrifuged at 800 g for 5 min. The supernatant was collected, aliquoted, and stored in 20% glycerin-PBS solution at − 80 °C until transplantation.

#### Recipient rats

To decrease the bacterial load in the recipient rats, they were treated with 1 ml of antibiotic cocktail consisting of vancomycin (50 mg/kg), neomycin (100 mg/kg), metronidazole (100 mg/kg), and amphotericin-B (1 mg/kg) once daily by oral gavage for 7 consecutive days. Their drinking water was also supplemented with ampicillin (1 g/L) [81]. All antibiotics were purchased from Solarbio Science & Technology Co., Ltd. (Beijing, China). Forty-eight hours after the antibiotic treatment, the recipient rats were treated with 1 ml donor gut microbiota supernatant by gavage, repeated daily. After surgery, FMT was conducted every other day until sacrifice. Feces from the recipient rats were collected for 16S rRNA gene sequencing before sacrifice to evaluate the rebuilt microbiome composition.

### SCFA supplementation

After undergoing surgery, the rats were given a mixture of SCFAs with 67.5 mM sodium acetate (S2889, Sigma), 25.9 mM sodium propionate (P1880, Sigma), and 40 mM sodium butyrate (303410, Sigma), or a salt-matched control solution (133.4 mM sodium chloride) (S9888, Sigma) in drinking water ad libitum continuously except for during the SPT [75].

### Fecal parameters measurement

FPM was performed as described previously [82]. In brief, the rats were individually housed, and given 4 h of habituation. Then, each rat was observed for 8 h with free access to food and water. Fecal pellets were collected and counted every 2 h. The fecal dimensions and weight of each rat were measured. Fecal water content was identified by comparing the weight of the pellets before and after drying (24 h at 60 °C).

### Intestinal motility test

To evaluate intestinal mobility and permeability, fluorescein isothiocyanate-dextran (FD4, #60842-46-8-FD4-1 G, Sigma-Aldrich, USA) was used according to previous studies [42, 83]. Briefly, the rats with a 24-h food deprivation were administered intragastrically with 40-kDa FD4 200 mg/kg dissolved in sterile saline. Three hours later, the rats were anesthetized via an intraperitoneal injection of chloral hydrate (10%, 3 mL/kg). The blood from the portal vein was collected and immediately centrifuged at 8228 g for 10 min to acquire the supernatant. Then blood FD4 concentration was quantified using a Multiskan Mk3 microplate reader (Thermo, USA) with a standard curve at an excitation wavelength of 488 nm. Meanwhile, the entire intestinal tract from stomach to colon was removed and imaged using a Caliper IVIS Lumina III (PerkinElmer, USA). For quantification of gastrointestinal motility, the whole gastrointestinal tract was divided into 14 segments and flushed with distilled water. Using a fluorescence plate reader (Promega), the fluorescence of the purified recovered flushing solution was measured, followed by normalization to blank controls, and then expressed as the percentage of fluorescence per segment.

### LPS challenge

LPS (L2630, Sigma) powder was dissolved in normal saline. After FMT treatment and behavioral tests, two groups of sham rats were given LPS (1 mg/kg) via intraperitoneal injection. Six hours later, the animals were sacrificed for analysis.

### Immunofluorescence, TUNEL, and immunohistochemistry staining

After fasting for 24 h, the rats were perfused intracardially with ice-cold saline and 4% paraformaldehyde under anesthetic. The brains and colons were harvested and immersed in 4% paraformaldehyde for after-fixing. After dehydrating the brain tissues with 20% sucrose and 30% sucrose in sequence, 30-μm coronal brain sections were prepared using a freezing microtome (HM525NX, ThermoFisher, USA). The brain sections were then washed with PBS and PBS+0.3% Triton, and blocked with 10% goat serum or donkey serum for 1 h, followed by overnight incubation (4 °C) with the following primary antibodies: mouse monoclonal anti-NeuN (1:1000, 66836-1-Ig; Proteintech, USA), rabbit polyclonal anti-MBP (1:1000, 10458-1-AP; Proteintech, USA), goat polyclonal anti-Iba-1 (1:1,000, ab5076; Abcam, England), and rabbit polyclonal anti-GPR41 (1:200, ab236654; Abcam, England). Then the sections were treated with the appropriate Alexa-Fluor-conjugated antibodies (1:1000; Jackson ImmunoResearch, USA) for 1 h at room temperature and coverslipped with DAPI-Fluoromount-G (Sigma, USA). TUNEL staining of brain sections was conducted using a TUNEL apoptosis assay kit (Beyotime, China) according to the manufacturer’s instructions. The colons were dehydrated and embedded in paraffin. Sections (5 μm thick) were stained with AB-PAS (Acmec, China). The images were analyzed using the ImageJ (version 1.8.0, NIH) software.

### Western blotting

The mitochondrial proteins in the hippocampal region were extracted from the fresh brain tissue using a tissue mitochondria isolation kit (Beyotime, China) according to the manufacturer’s instructions. To acquire the total protein, the hippocampal tissue was homogenized in cold RIPA buffer (9806; Cell Signaling Technology, USA), containing 1 mmol/L phenylmethylsulfonyl fluoride and a protease inhibitor cocktail (1: 20, Cat# P-2714; Sigma, USA), followed by centrifugation at 12000 rpm for 15 min at 4 °C and collection of the supernatant. Then the sample proteins were quantified using a BCA protein assay kit (Beyotime, China). The same mass of protein (approximately 30–50 μg depending on the protein content in the tissue) was loaded on each well of a gel. After separation on sodium dodecyl sulfate gels of appropriate concentrations, the proteins were transferred to polyvinylidene difluoride (PVDF) membranes (Merck, Germany). The PVDF membranes were blocked with 5% nonfat dried milk powder/Tris-buffered saline Tween-20 for 1 h and probed with primary antibodies. The primary antibodies used included rabbit polyclonal anti-caspase 3 (1:1,000, 19677-1-AP; Proteintech, USA), rabbit polyclonal anti-Bcl 2 (1:1,000, 12789-1-AP; Proteintech, USA), rabbit polyclonal anti-Bax (1:1,000, 50599-2-Ig; Proteintech, USA), rabbit polyclonal anti-Bcl-XL (1:1,000, 26967-1-AP; Proteintech, USA), rabbit monoclonal anti-cytochrome c oxidase subunit IV isoform (COXIV) (1:5,000, 66110-1-Ig; Proteintech, USA), rabbit monoclonal anti-phospho-Erk1/2 (1:1,000, #4370; Cell Signaling Technology, USA), rabbit monoclonal anti-Erk1/2 (1:1,000, #4695; Cell Signaling Technology, USA), rabbit monoclonal anti-NF-κB p65 (1:1,000, #8242; Cell Signaling Technology, USA), rabbit monoclonal anti-CD16 (1:1,000, ab211151; Abcam, England), and mouse monoclonal anti-β-Actin (1:5,000, #3700; Cell Signaling Technology, USA). After incubation overnight at 4 °C with primary antibodies, the membranes were incubated with peroxidase-conjugated secondary antibodies (1:5,000; Proteintech, USA) and visualized using the ChemiDoc MP System (Bio-Rad, USA). The band intensity quantification was performed using the ImageJ (version 1.8.0, NIH) software.

### Isolation of total RNA and real-time PCR analysis

The total RNA from the tissue samples was isolated using TRIzol reagent (Invitrogen; Thermo Fisher Scientific. Inc.). cDNA synthesis for mRNA was carried out using a PrimeScript RT Reagent Kit (Takara, Shiga, Japan). After the RT reaction, 2 μL of cDNA was used for subsequent qPCR with SYBR Green dye (Sigma-Aldrich, USA), conducted with a 7500 Real-Time PCR System (Applied Biosystems; Thermo Fisher Scientific, Inc.). The PCR conditions were: 40 cycles of 95 °C for 30 s, 60 °C for 34 s, and 72 °C for 30 s. All of the primer sequences are listed in Additional file 4 (Table S1). The relative expression levels of the candidate genes were analyzed using the 2^−ΔΔCt^ method.

### SCFA quantification

SCFAs from hippocampus samples were analyzed as previously described, with some modifications [84]. In short, 50-mg samples of hippocampal tissue were vortexed in a phosphoric acid (0.5% v/v) solution for 10 s. Following homogenization for 4 min at 40 Hz, the samples were treated with ultrasonic waves for 5 min at 4 °C. After 10 min of centrifugation at 12,000 r/min at 4 °C, the supernatant was collected and analyzed using GC-MS (7890B5977B; Agilent Technologies Inc.) on a silica capillary column (HP-FFAP, Agilent J&W). The main conditions for mass spectrometry are listed in Additional file 4 (Table S2).

### Statistical analysis

The data were analyzed using SPSS software (Version 20, Chicago, USA) and presented as mean ± standard error of the mean (SEM). The normality and variance homogeneity of the data were confirmed before each parametric test. Comparisons of continuous variables were performed using the two-tailed Student’s *t* test, or one-way analysis of variance (ANOVA) followed by Tukey’s multiple comparisons test. A *p* value of < 0.05 was considered to represent statistical significance.

### Supplementary Information

**Additional file 1: Figure S1**. Effects of BCCAO on body weight (A), defecation frequency (B), length of fecal pellets (C), fecal wet weight (D), fecal dry weight (E), and fecal water content (F) in rats (*n* = 8 Sham, *n* = 10 BCCAO). The data represent the mean ± SEM, *p* < 0.05 was set as the threshold for significance. * *p* < 0.05 compared to the sham group. **Figure S2**. Effects of BCCAO on gut function and microbial ecology. A One of the BCCAO rats was excluded from the intestinal motility test due to intestinal obstruction. B The structure of the gut microbiota at the phylum level (*n* = 15/group). **Figure S3**. Overall representation of bacterial profiles in sham and BCCAO rats by linear discriminant effect size (LEfSe) analysis (*n* = 15/group). **Figure S4**. A heatmap demonstrating the difference in gut microbiota profile at the family level between the sham and BCCAO groups (*n* = 15/group). **Figure S5**. A heatmap demonstrating the difference in gut microbiota profile at the genus level between the sham and BCCAO groups (*n* = 15/group). **Figure S6**. Effects of BCCAO on hippocampal neuron, white matter injury and microglial activation. A Bar plots showing the number of NeuN+ cells in different areas of the hippocampus, namaly cornu ammonis (CA) 1, CA2, CA3, and dentate gyrus (DG) (*n* = 5 Sham, *n* = 6 BCCAO). B Immunofluorescence staining of myelin basic protein (MBP). Scale bar, 1 mm. C Representative images of the medial corpus callosum (CC), paramedian CC, internal capsule (IC), and caudoputamen regions and D bar plots showing the mean fluorescence intensity (*n* = 5 Sham, *n* = 6 BCCAO). Scale bar, 50 μm. E mRNA levels of *Iba-1*, microglial pro-inflammatory marker (*CD16*), and anti-inflammatory marker (*CD206*) in the hippocampus at day 36 post BCCAO (*n* = 6/group). The data represent the mean ± SEM, *p* < 0.05 was set as the threshold for significance. * *p* < 0.05, ** *p* < 0.01 compared to the sham group. **Figure S7**. Fecal microbiota transplantation successfully rebuilds distinct gut microbiota composition. A The structure of gut microbiota at the phylum level successfully reestablished by FMT (*n* = 10/group). B Bar plots showing the relative abundance of f_*Ruminococcaceae* and f_*Prevotellaceae* at the family level and g_*Bifidobacterium*, g_*Bacteroides*, g_*Clostridium*, g_*Blautia*, g_*Roseburia*, g_*Coprococcus*, g_*Odoribacter*, and g_*Dorea* at the genus level (*n* = 10/group). S+FMT(B), sham rats received BCCAO-rat-derived fecal microbiota transplantation. S+FMT(S), sham rats received sham-rat-derived fecal microbiota transplantation. B+FMT(B), BCCAO rats received BCCAO-rat-derived fecal microbiota transplantation. B+FMT(S), BCCAO rats received sham-rat-derived fecal microbiota transplantation. The data represent the mean ± SEM. *p* < 0.05 was set as the threshold for significance. * *p* < 0.05, ** *p* < 0.01, *** *p* < 0.001 compared to the S+FMT(B) group. ^&^*p* < 0.05 compared to the S+FMT(S) group. ^#^*p* < 0.05, ## *p* < 0.01 compared to the B+FMT(B) group. **Figure S8**. Effects of FMT on body weight (A), defecation frequency (B), length of fecal pellets (C), fecal wet weight (D), fecal dry weight (E), and fecal water content (F) in recipient rats (*n* = 10 S+FMT(B) and S+FMT(S), *n* = 14 B+FMT (B) and B+FMT(S)). The data represent the mean ± SEM, *p* < 0.05 was set as the threshold for significance. ** *p* < 0.01, *** *p* < 0.001 compared to the S+FMT(B) group. ^&&&^*p* < 0.001 compared to the S+FMT(S) group. ^#^*p* < 0.05 compared to the B+FMT(B) group. **Figure S9**. Effects of FMT on the number of NeuN+ cells in different areas of the hippocampus, namaly (A) CA 1, (B) CA2, (C) CA3, and (D) DG (*n* = 6 S+FMT(B) and S+FMT(S), *n* = 8 B+FMT(B) and B+FMT(S)). The data represent the mean ± SEM, *p* < 0.05 was set as the threshold for significance. ** *p* < 0.01, *** *p* < 0.001 compared to the S+FMT(B) group. ^&^*p* < 0.05, ^&&&^*p* < 0.001 compared to the S+FMT(S) group. ^#^*p* < 0.05 compared to the B+FMT(B) group. **Figure S10**. Effects of FMT on white matter injury after BCCAO. A Representative immunofluorescence staining images of MBP in the medial CC, paramedian CC, IC, and caudoputamen regions. Scale bar, 50 μm. B Bar plots showing the mean fluorescent intensity (*n* = 6/group). The data represent the mean ± SEM, *p* < 0.05 was set as the threshold for significance. ** *p* < 0.01, *** *p* < 0.001 compared to the S+FMT(B) group. ^&^*p* < 0.05, ^&&^*p* < 0.01 compared to the S+FMT(S) group. **Figure S11**. Effects of FMT on SCFA levels in the hippocampus of recipient rats, including acetic acid (A), propionic acid (B), isobutyric acid (C), butyric acid (D), isovaleric acid (E), valeric acid (F), and hexanoic acid (G) (*n* = 8/group). The data represent the mean ± SEM, *p* < 0.05 was set as the threshold for significance. * *p* < 0.05 compared to the S+FMT(B) group. ^#^*p* < 0.05 compared to the B+FMT(B) group. **Figure S12**. Immunofluorescence staining of the GPR41-a receptor of SCFAs with hippocampal neurons (A) and microglia (B). Arrows indicate the expression of GPR-41 on microglia. Scale bar, 20 μm. **Figure S13**. Effects of long-term SCFA supplementation on body weight (A), defecation frequency (B), length of fecal pellets (C), fecal wet weight (D), fecal dry weight (E), and fecal water content (F) in rats (*n* = 8 S+NaCl and S+SCFA, *n* = 12 B+NaCl and B+SCFA). S+NaCl, sham rats treated with sodium chloride (NaCl). S+SCFA, sham rats that received short chain fatty acids (SCFAs) (acetate, propionate and butyrate) supplementation. B+NaCl, BCCAO rats treated with NaCl. B+SCFA, BCCAO rats received SCFAs supplementation. The data represent the mean ± SEM, *p* < 0.05 was set as the threshold for significance. ** *p* < 0.01 compared to the S+NaCl group. ^&&^*p* < 0.01 compared to the S+SCFA group. **Figure S14**. Effects of long-term SCFA supplementation on the number of NeuN+ cells in different areas of the hippocampus, namaly (A) CA 1, (B) CA2, (C) CA3, and (D) DG (*n* = 6). The data represent the mean ± SEM, *p* < 0.05 was set as the threshold for significance. * *p* < 0.05, ** *p* < 0.01, *** *p* < 0.001 compared to the S+NaCl group. ^&^*p* < 0.05, ^&&^*p* < 0.01 compared to the S+SCFA group. ^#^*p* < 0.05 compared to the B+NaCl 37 d group.

**Additional file 2.** The original data of 16S rRNA gene sequencing to investigate the gut microbiota composition of sham rats and BCCAO rats. (XLS 213 kb)

**Additional file 3.** The original data of 16S rRNA gene sequencing to investigate the rebuilt gut microbiota composition after FMT treatment. (XLS 12860 kb)

**Additional file 4: Table S1**. The primer sequences for real-time PCR. **Table S2**. The main mass spectrometry conditions for SCFA quantification.

## Data Availability

All data generated or analyzed during this study are included in this published article and its supplementary information files.

## Abbreviations

CCH: Chronic cerebral hypoperfusion
CNS: Central nervous system
SCFAs: Short-chain fatty acids
BCCAO: Bilateral common carotid artery occlusion
FMT: Fecal microbiota transplantation
AD: Alzheimer’s disease
VaD: Vascular dementia
VCI: Vascular cognitive impairment
CCH: Chronic cerebral hypoperfusion
Aβ: β-Amyloid
APP: Aβ precursor protein
OFT: Open-field test
MWM: Morris water maze test
SPT: Sucrose preference test
TST: Tail suspension test
FST: Forced swim test
FPM: Fecal parameters measurement
IMT: Intestinal motility test
FD4: Fluorescein isothiocyanate-dextran
Muc: Mucins
AB-PAS: Alcian blue and periodic acid-Schiff
TNF-α: Tumor necrosis factor-alpha
IL-1β: Interleukin-1 beta
rRNA: Ribosomal RNA
PCoA: Principal coordinate analysis
LEfSe: Linear discriminant analysis coupled with effect size
CA: Cornu ammonis
DG: Dentate gyrus
MBP: Myelin basic protein
Iba-1: Ionized calcium-binding adaptor molecule-1
GC-MS: Gas chromatography-mass spectrometry
Bcl2: B cell lymphoma 2
Bax: B cell lymphoma 2-associated X regulator
Bcl-XL: B cell lymphoma 2-like 1 L isoform
COXIV: Cytochrome c oxidase subunit IV isoform
CC3: Cleaved caspase 3
GPR41: G protein-coupled receptor 41
pErk1/2: Phospho-p44/42 mitogen-activated protein kinase
Erk1/2: p44/42 mitogen-activated protein kinase
LPS: Lipopolysaccharide
CD16: Fc RII/III receptor
NF-κB: Nuclear factor kappa beta
MMD: Moyamoya disease
CCA: Common carotid artery
QIIME: Quantitative Insights Into Microbial Ecology
OUT: Operational taxonomic units
ASVs: Amplicon sequence variants
PBS: Phosphate-buffered saline solution
PVDF: Polyvinylidene difluoride
SEM: Mean ± standard error of the mean
ANOVA: One-way analysis of variance
