# Correction to: The microbiota-gut-brain axis participates in chronic cerebral hypoperfusion by disrupting the metabolism of short-chain fatty acids

**DOI:** 10.1186/s40168-022-01277-0

**Published:** 2022-05-04

**Authors:** Weiping Xiao, Jiabin Su, Xinjie Gao, Heng Yang, Ruiyuan Weng, Wei Ni, Yuxiang Gu

**Affiliations:** 1 Department of Neurosurgery, Huashan Hospital, Fudan University, Shanghai, 200040 China; 2 Institute of Neurosurgery, Fudan University, Shanghai, 200052 China; 3 Shanghai Key Laboratory of Brain Function and Restoration and Neural Regeneration, Shanghai, 200052 China

**Correction to: Microbiome 10, 62 (2022)**

**https://doi.org/10.1186/s40168-022-01255-6**

Following the publication of the original article [1], the author reported that there are grey boxes in Figs. 2K, 3D, and 4D. The correct figures are provided here.

**Fig. 2:**
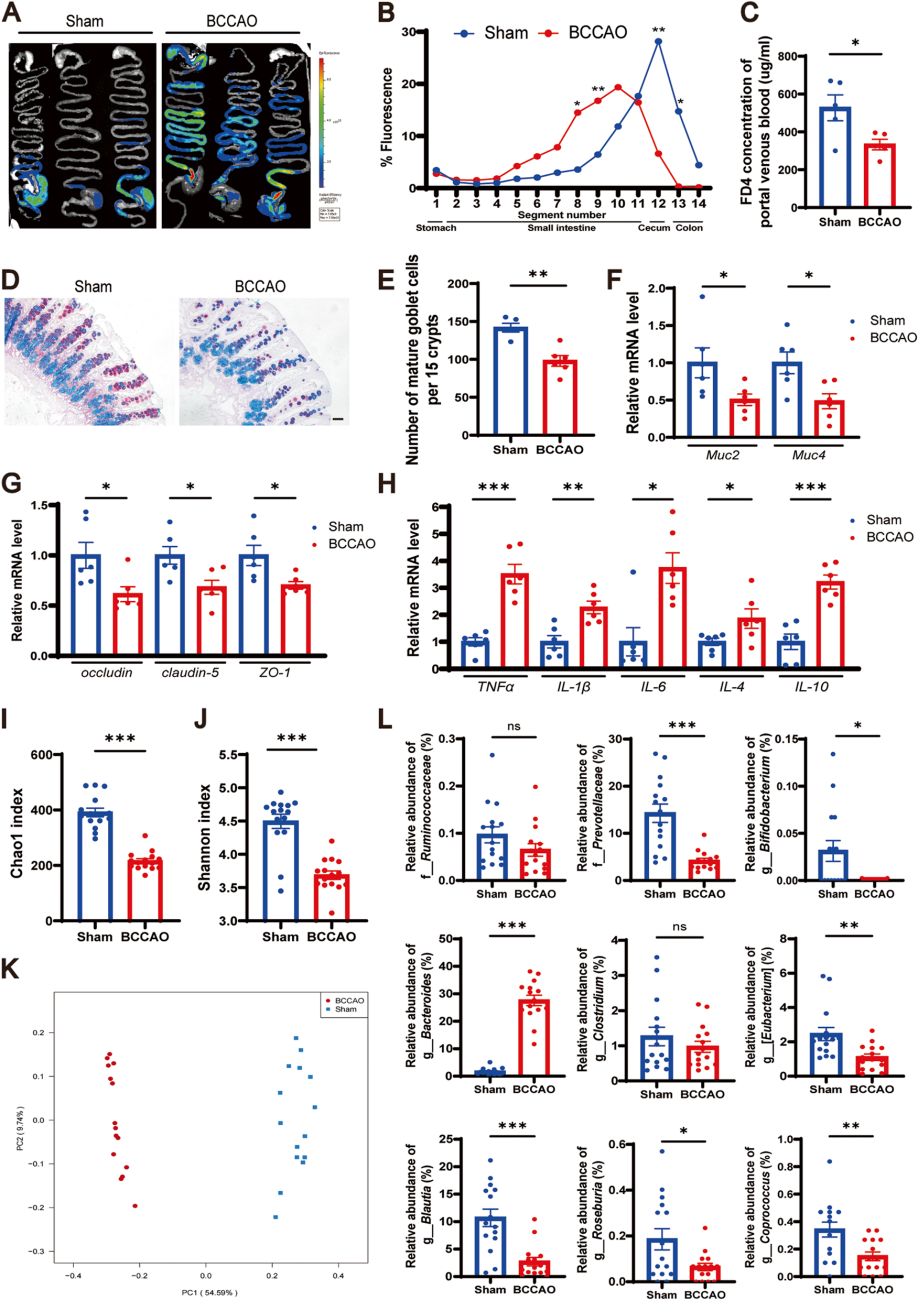
.

**Fig. 3:**
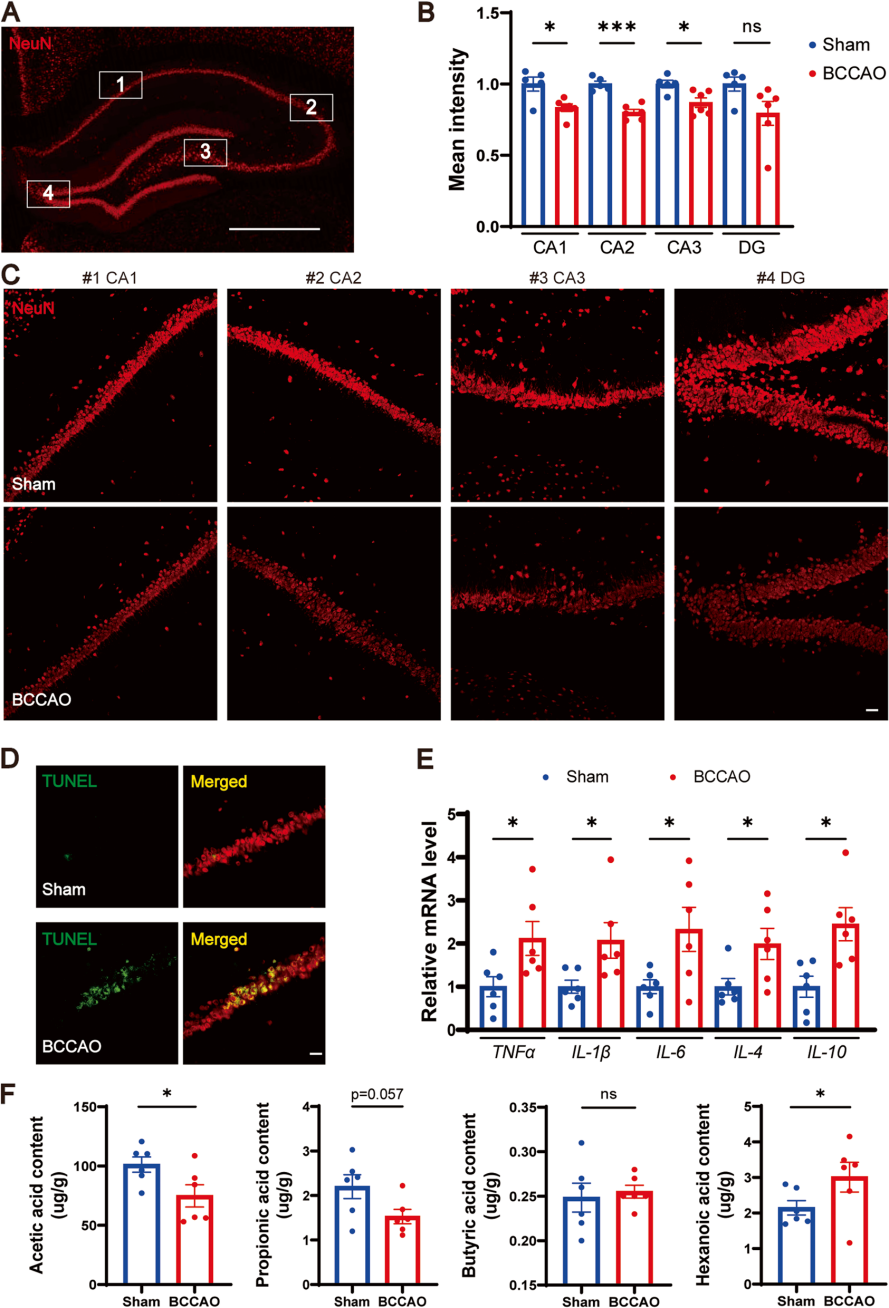
.

**Fig. 4:**
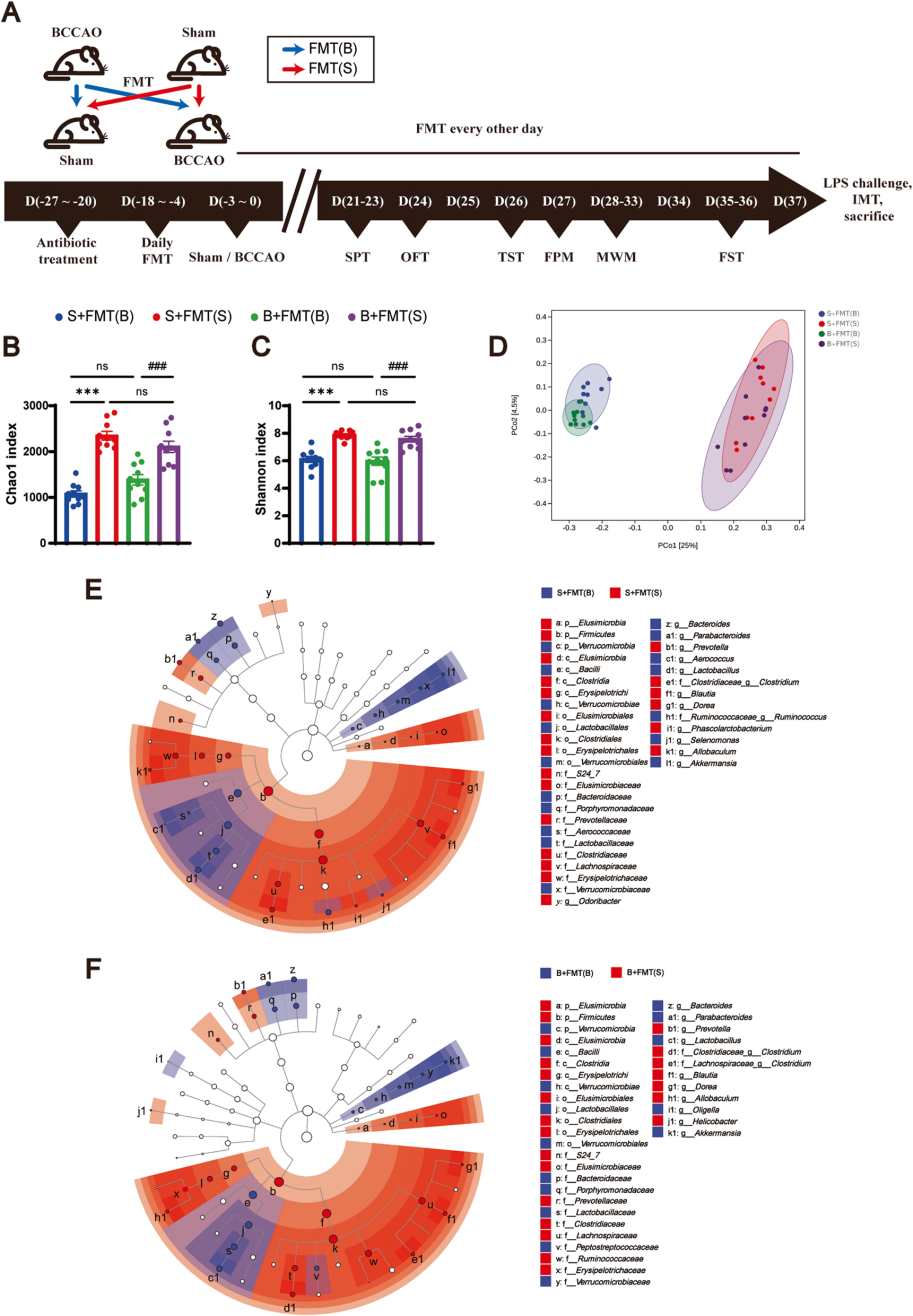
.

The original article has been updated.

## References

[1] Xiao W, Su J, Gao X (2022). The microbiota-gut-brain axis participates in chronic cerebral hypoperfusion by disrupting the metabolism of short-chain fatty acids. Microbiome.

