# Correction: The microbiota-gut-brain axis participates in chronic cerebral hypoperfusion by disrupting the metabolism of short-chain fatty acids

**DOI:** 10.1186/s40168-024-01842-9

**Published:** 2024-06-04

**Authors:** Weiping Xiao, Jiabin Su, Xinjie Gao, Heng Yang, Ruiyuan Weng, Wei Ni, Yuxiang Gu

**Affiliations:** 1 Department of Neurosurgery, Huashan Hospital, Fudan University, Shanghai, 200040 China; 2 Institute of Neurosurgery, Fudan University, Shanghai, 200052 China; 3 Shanghai Key Laboratory of Brain Function and Restoration and Neural Regeneration, Shanghai, 200052 China

**Correction** **:** **Microbiome 10, 62 (2022)**

**https://doi.org/10.1186/s40168-022-01255-6**

Following publication of the original article [1], the author reported that in Figure 9D, the first image is a duplicate of the second image. The correct image is below.


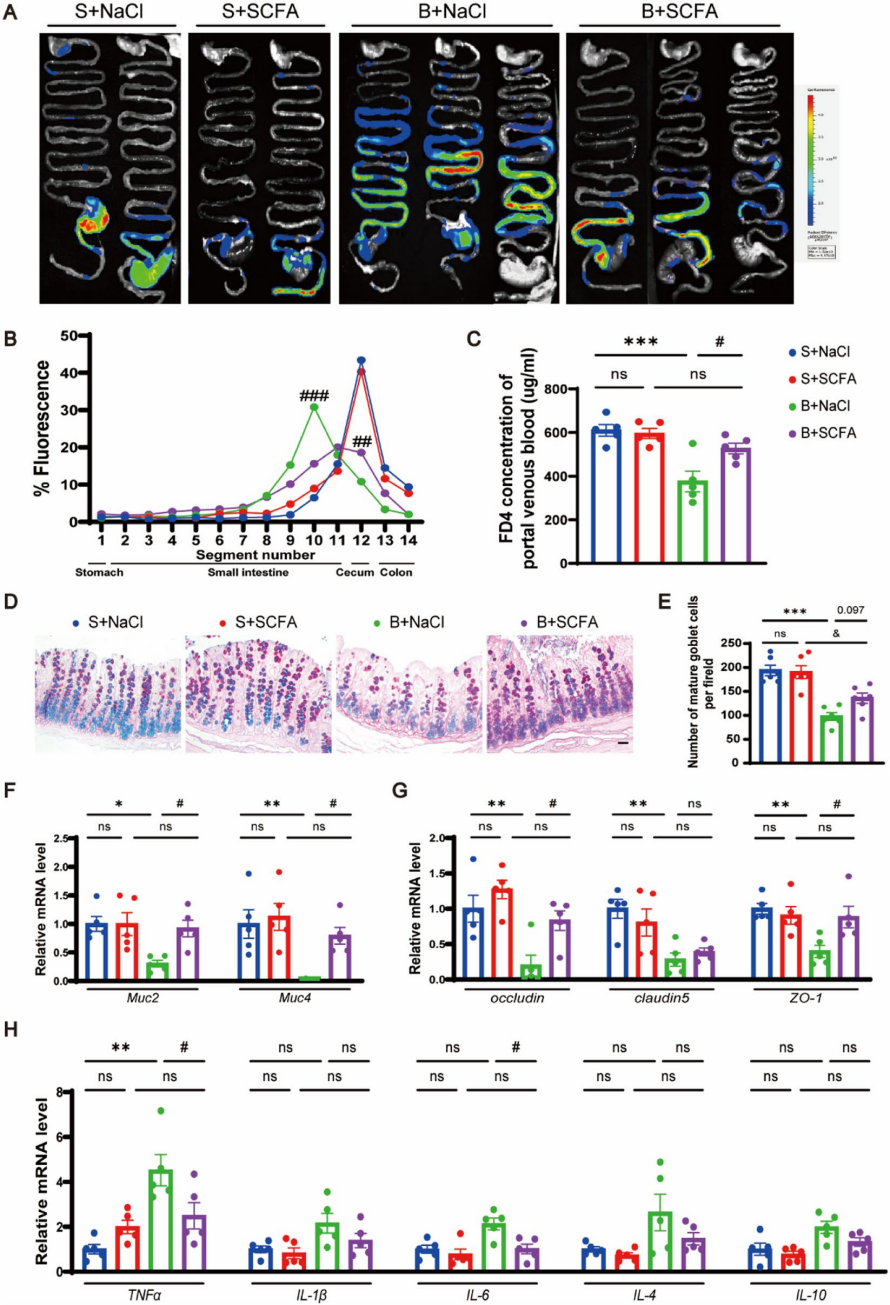


The original article has been updated.
