# Supplementary material for: The microbiota-gut-brain axis participates in chronic cerebral hypoperfusion by disrupting the metabolism of short-chain fatty acids
Source: Microbiome. 2022 Apr 17;10:62. doi: 10.1186/s40168-022-01255-6 (PMC9013454; doi:10.1186/s40168-022-01255-6)
Supplement: Supplementary file 2 — Additional file 1: Figure S1. Effects of BCCAO on body weight (A), defecation frequency (B), length of fecal pellets (C), fecal wet weight (D), fecal dry weight (E), and fecal water content (F) in rats (n = 8 Sham, n = 10 BCCAO). The data represent the mean ± SEM, p < 0.05 was set as the threshold for significance. * p < 0.05 compared to the sham group. Figure S2. Effects of BCCAO on gut function and microbial ecology. A One of the BCCAO rats was excluded from the intestinal motility test due to intestinal obstruction. B The structure of the gut microbiota at the phylum level (n = 15/group). Figure S3. Overall representation of bacterial profiles in sham and BCCAO rats by linear discriminant effect size (LEfSe) analysis (n = 15/group). Figure S4. A heatmap demonstrating the difference in gut microbiota profile at the family level between the sham and BCCAO groups (n = 15/group). Figure S5. A heatmap demonstrating the difference in gut microbiota profile at the genus level between the sham and BCCAO groups (n = 15/group). Figure S6. Effects of BCCAO on hippocampal neuron, white matter injury and microglial activation. A Bar plots showing the number of NeuN+ cells in different areas of the hippocampus, namaly cornu ammonis (CA) 1, CA2, CA3, and dentate gyrus (DG) (n = 5 Sham, n = 6 BCCAO). B Immunofluorescence staining of myelin basic protein (MBP). Scale bar, 1 mm. C Representative images of the medial corpus callosum (CC), paramedian CC, internal capsule (IC), and caudoputamen regions and D bar plots showing the mean fluorescence intensity (n = 5 Sham, n = 6 BCCAO). Scale bar, 50 μm. E mRNA levels of Iba-1, microglial pro-inflammatory marker (CD16), and anti-inflammatory marker (CD206) in the hippocampus at day 36 post BCCAO (n = 6/group). The data represent the mean ± SEM, p < 0.05 was set as the threshold for significance. * p < 0.05, ** p < 0.01 compared to the sham group. Figure S7. Fecal microbiota transplantation successfully rebuilds distinct gut microbiota c [file 40168_2022_1255_MOESM2_ESM.pdf]

Fig. S1

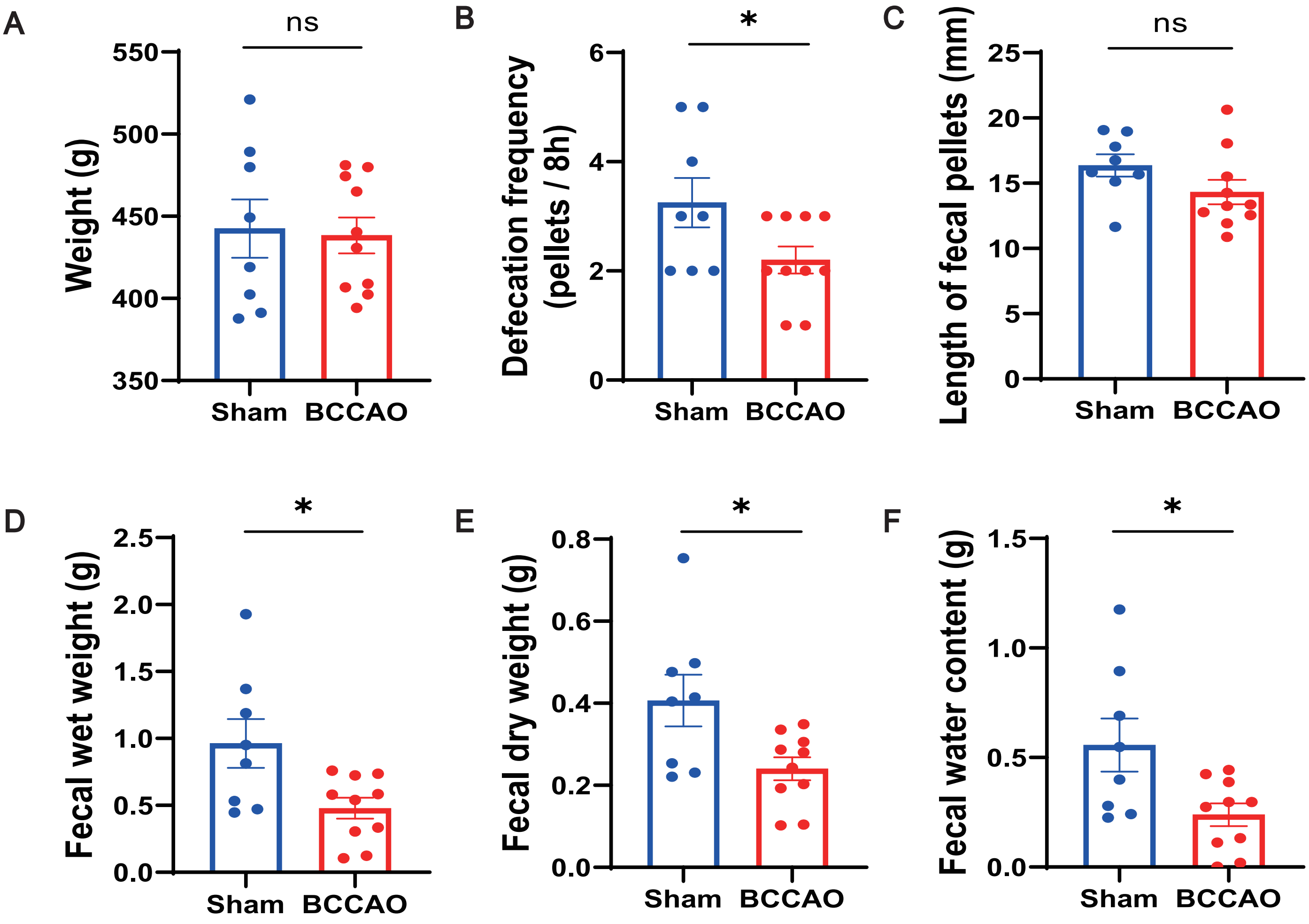

Fig. S1 Effects of BCCAO on body weight (**A**), defecation frequency (**B**), length of fecal pellets (**C**), fecal wet weight (**D**), fecal dry weight (**E**), and fecal water content (**F**) in rats (n = 8 Sham, n = 10 BCCAO). The data represent the mean ± SEM, $p < 0.05$ was set as the threshold for significance. * $p < 0.05$ compared to the sham group.

Fig. S2

A

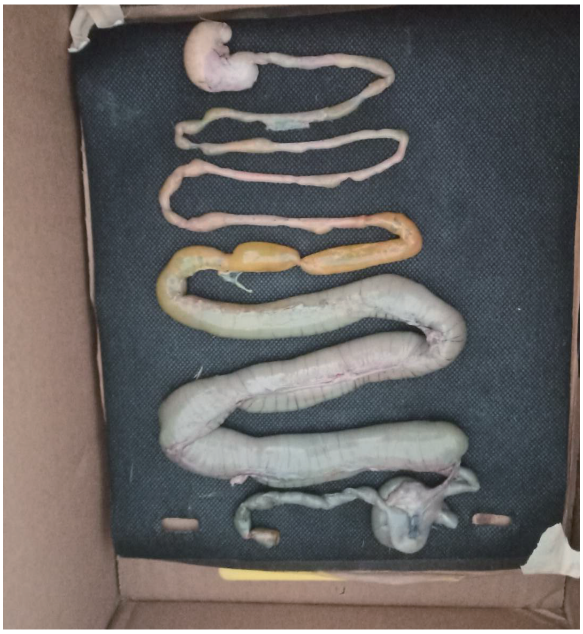

B

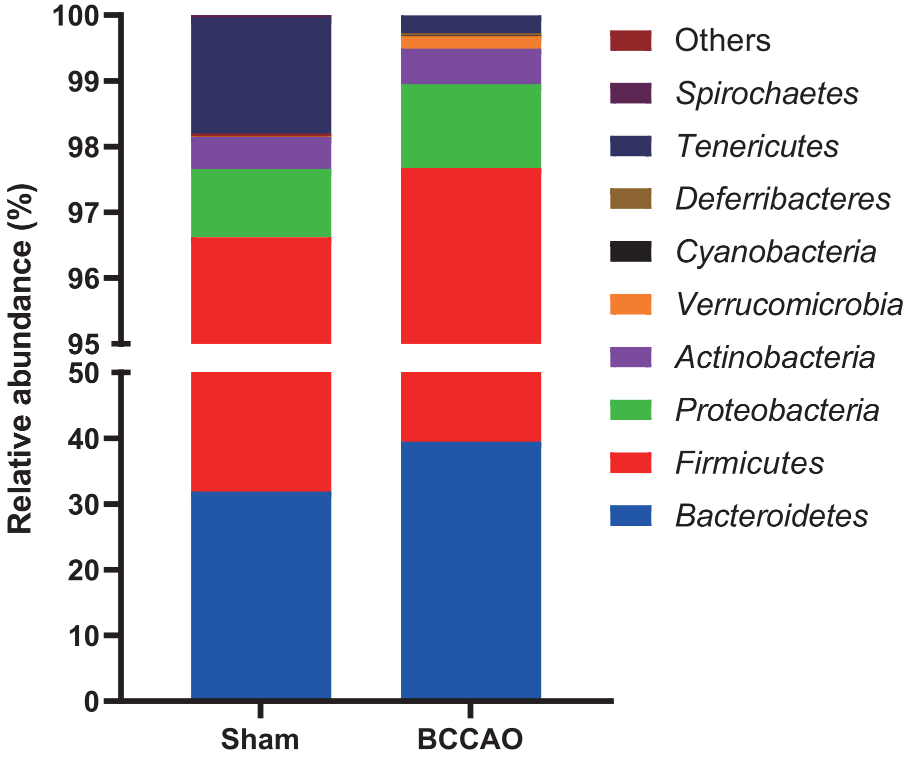

Fig. S2 Effects of BCCAO on gut function and microbial ecology. **A** One of the BCCAO rats was excluded from the intestinal motility test due to intestinal obstruction. **B** The structure of the gut microbiota at the phylum level (n = 15/group).

Fig. S3

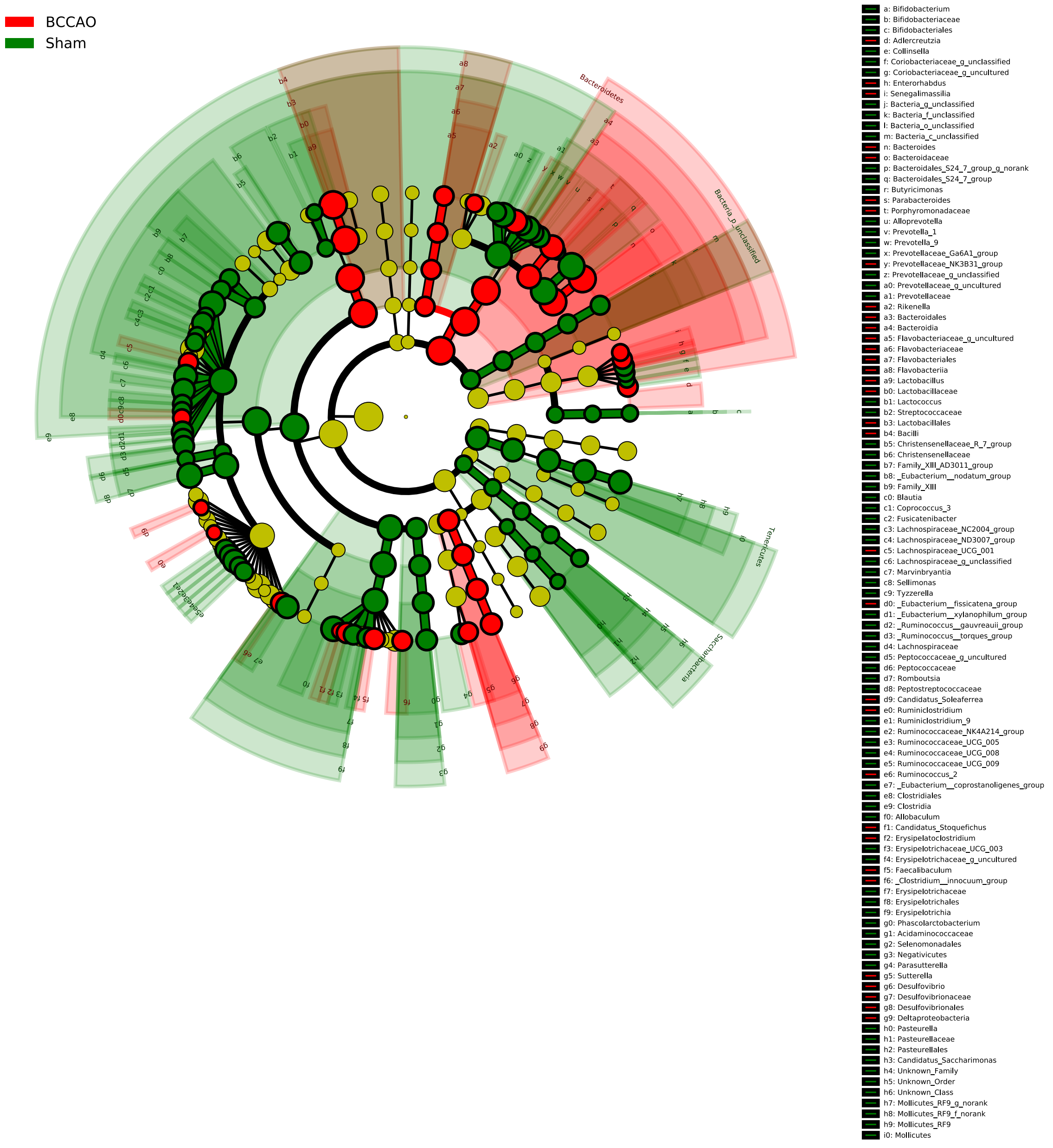

Fig. S3 Overall representation of bacterial profiles in the sham and BCCAO rats by linear discriminant effect size (LEfSe) analysis (n = 15/group).

Fig.S4

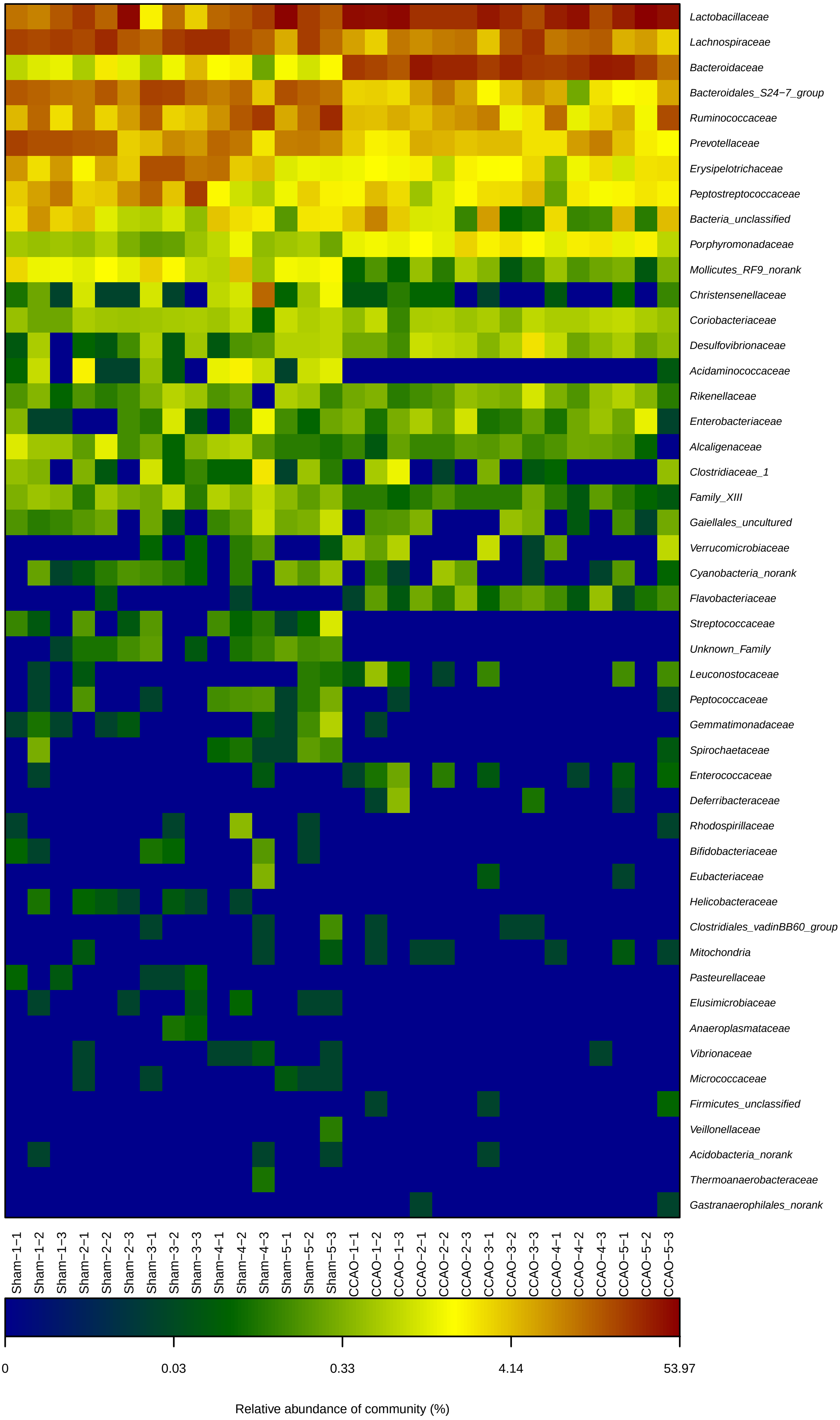

Fig. S4 A heatmap demonstrating the gut microbiota profile at the family level between the sham and BCCAO groups (n = 15/ group).

Fig.S5

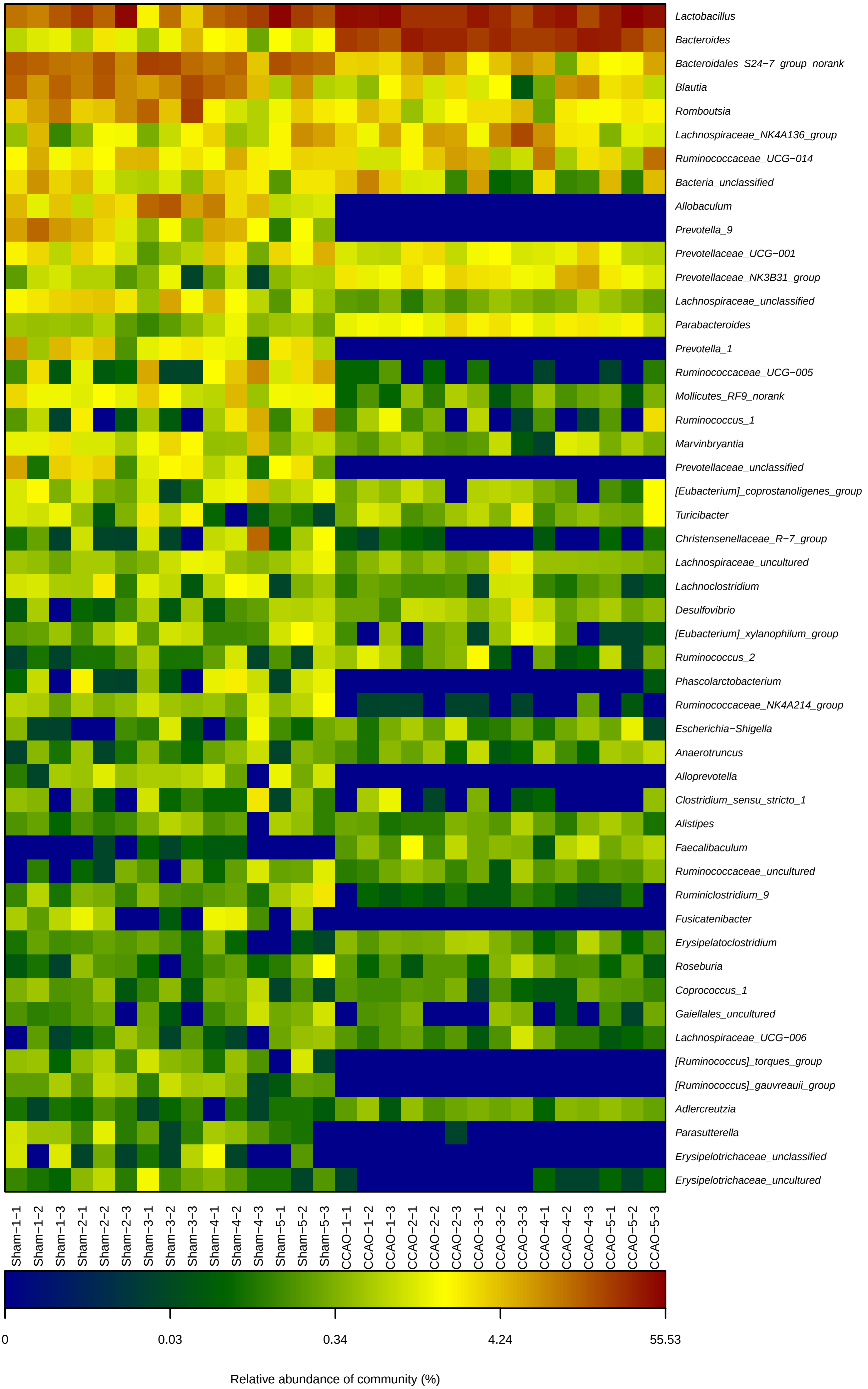

Fig. S5 A heatmap demonstrating the gut microbiota profile at the genus level between the sham and BCCAO groups (n = 15/group).

Fig. S6

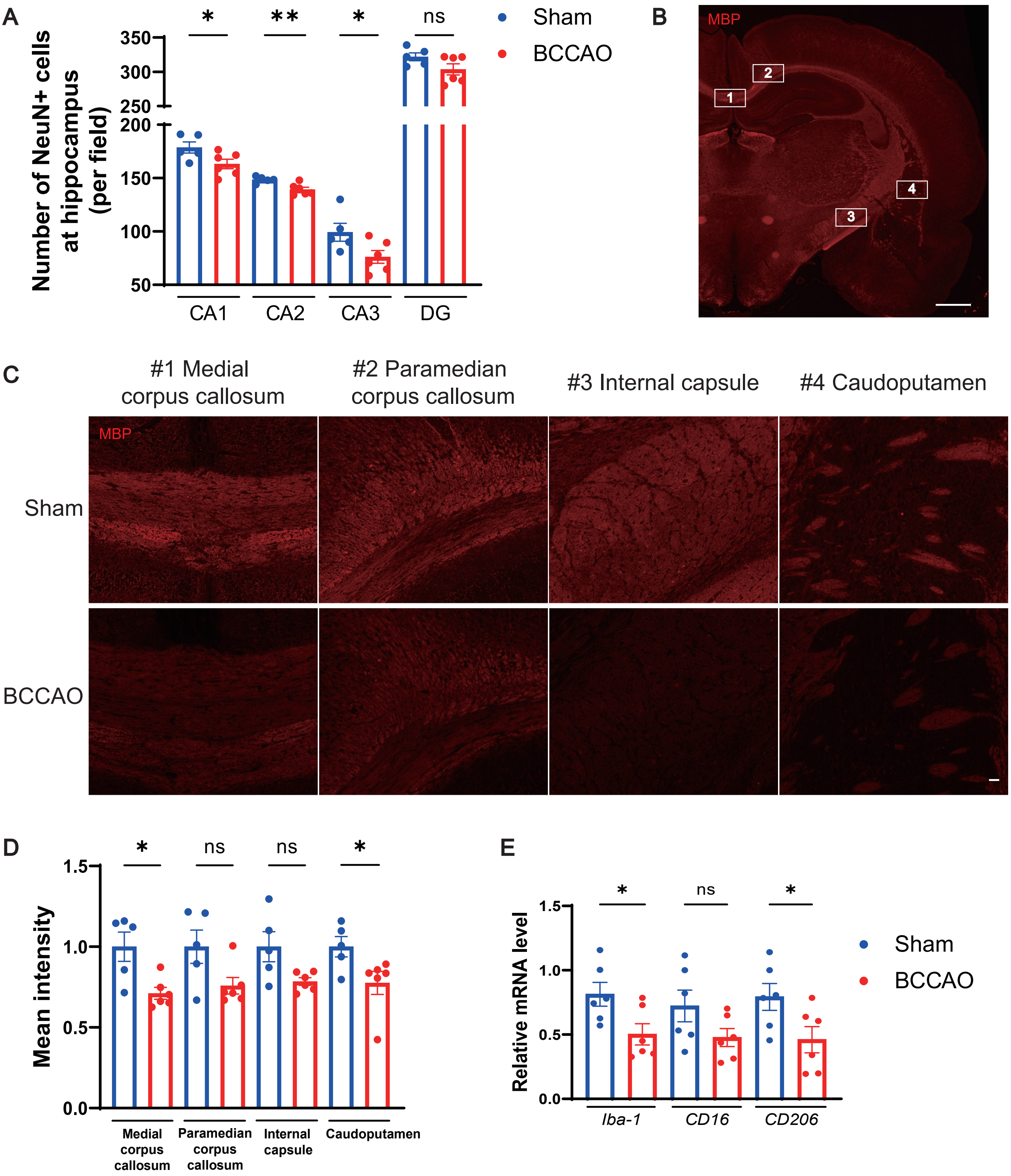

Fig. S6 Effects of BCCAO on hippocampal neuron, white matter injury and microglial activation. **A** Bar plots showing the number of NeuN+ cells in different areas of the hippocampus, namaly cornu ammonis (CA) 1, CA2, CA3, and dentate gyrus (DG) (n = 5 Sham, n = 6 BCCAO). **B** Immunofluorescence staining of myelin basic protein (MBP). Scale bar, 1 mm. **C** Representative images of the medial corpus callosum (CC), paramedian CC, internal capsule (IC), and caudoputamen regions and **D** bar plots showing the mean fluorescence intensity (n = 5 Sham, n = 6 BCCAO). Scale bar, 50 μm. **E** mRNA levels of Iba-1, microglial pro-inflammatory marker (*CD16*), and anti-inflammatory marker (*CD206*) in the hippocampus at day 36 post BCCAO (n = 6/group). The data represent the mean ± SEM, $p < 0.05$ was set as the threshold for significance. * $p < 0.05$, ** $p < 0.01$ compared to the sham group.

Fig. S7

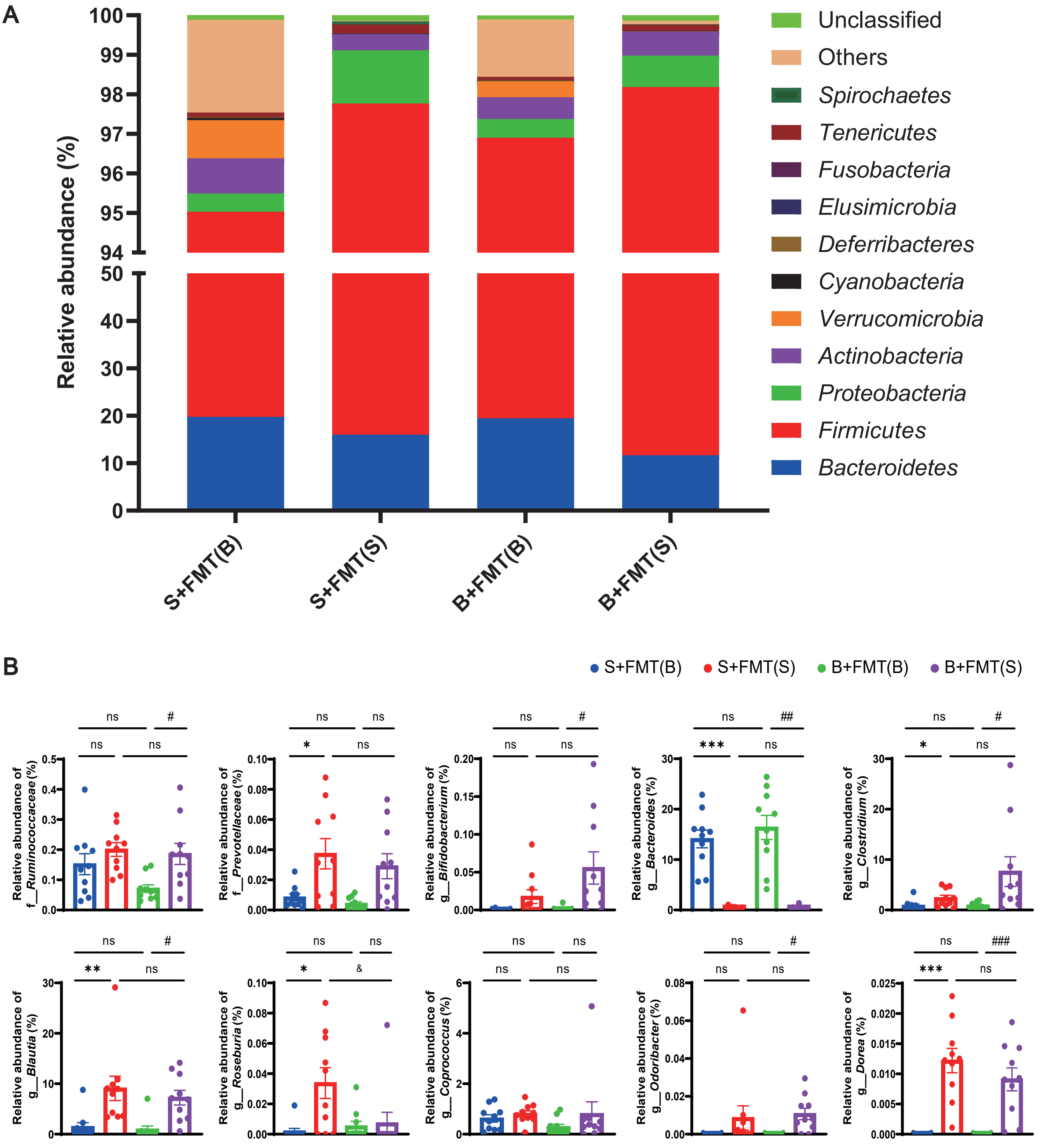

Fig. S7 Fecal microbiota transplantation successfully rebuilds distinct gut microbiota composition. **A** The structure of gut microbiota at the phylum level successfully reestablished by FMT (n = 10/group). **B** Bar plots showing the relative abundance of f_*Ruminococcaceae* and f_*Prevotellaceae* at the family level and g_*Bifidobacterium*, g_*Bacteroides*, g_*Clostridium*, g_*Blautia*, g_*Roseburia*, g_*Coprococcus*, and g_*Dorea* at the genus level (n = 10/group). S+FMT(B), sham rats received BCCAO-rat-derived fecal microbiota transplantation. S+FMT(S), sham rats received sham-rat-derived fecal microbiota transplantation. B+FMT(B), BCCAO rats received BCCAO-rat-derived fecal microbiota transplantation. B+FMT(S), BCCAO rats received sham-rat-derived fecal microbiota transplantation. The data represent the mean ± SEM. $p < 0.05$ was set as the threshold for significance. * $p < 0.05$, ** $p < 0.01$, *** $p < 0.001$ compared to the S+FMT(B) group. & $p < 0.05$ compared to the S+FMT(S) group. # $p < 0.05$, ## $p < 0.01$ compared to the B+FMT(B) group.

Fig. S8

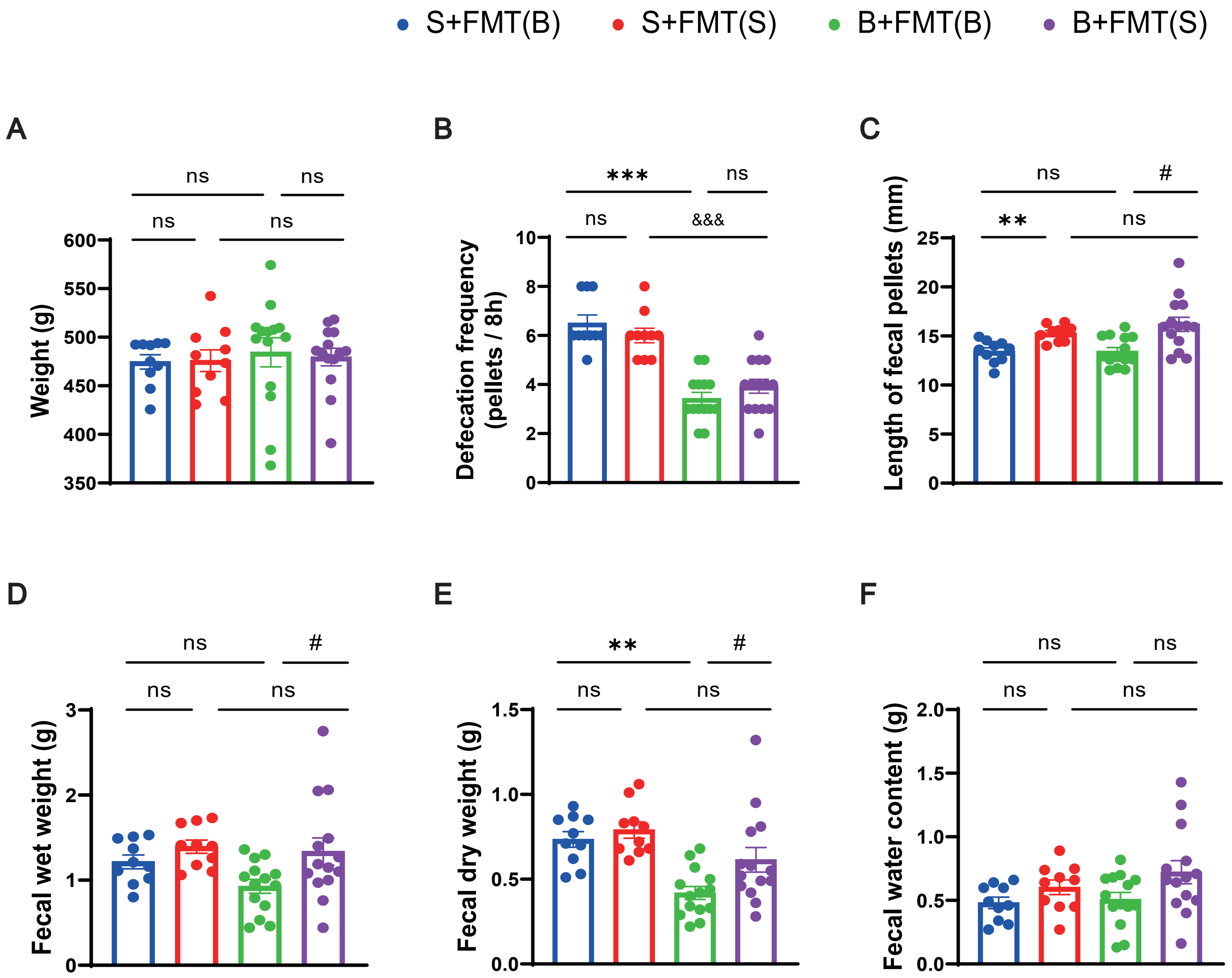

Fig. S8 Effects of FMT on body weight (**A**), defecation frequency (**B**), length of fecal pellets (**C**), fecal wet weight (**D**), fecal dry weight (**E**), and fecal water content (**F**) in recipient rats (n = 10 S+FMT(B) and S+FMT(S), n = 14 B+FMT(B) and B+FMT(S)). The data represent the mean ± SEM, $p < 0.05$ was set as the threshold for significance. ** $p < 0.01$, *** $p < 0.001$ compared to the S+FMT(B) group. &&& $p < 0.001$ compared to the S+FMT(S) group. # $p < 0.05$ compared to the B+FMT(B) group.

Fig. S9

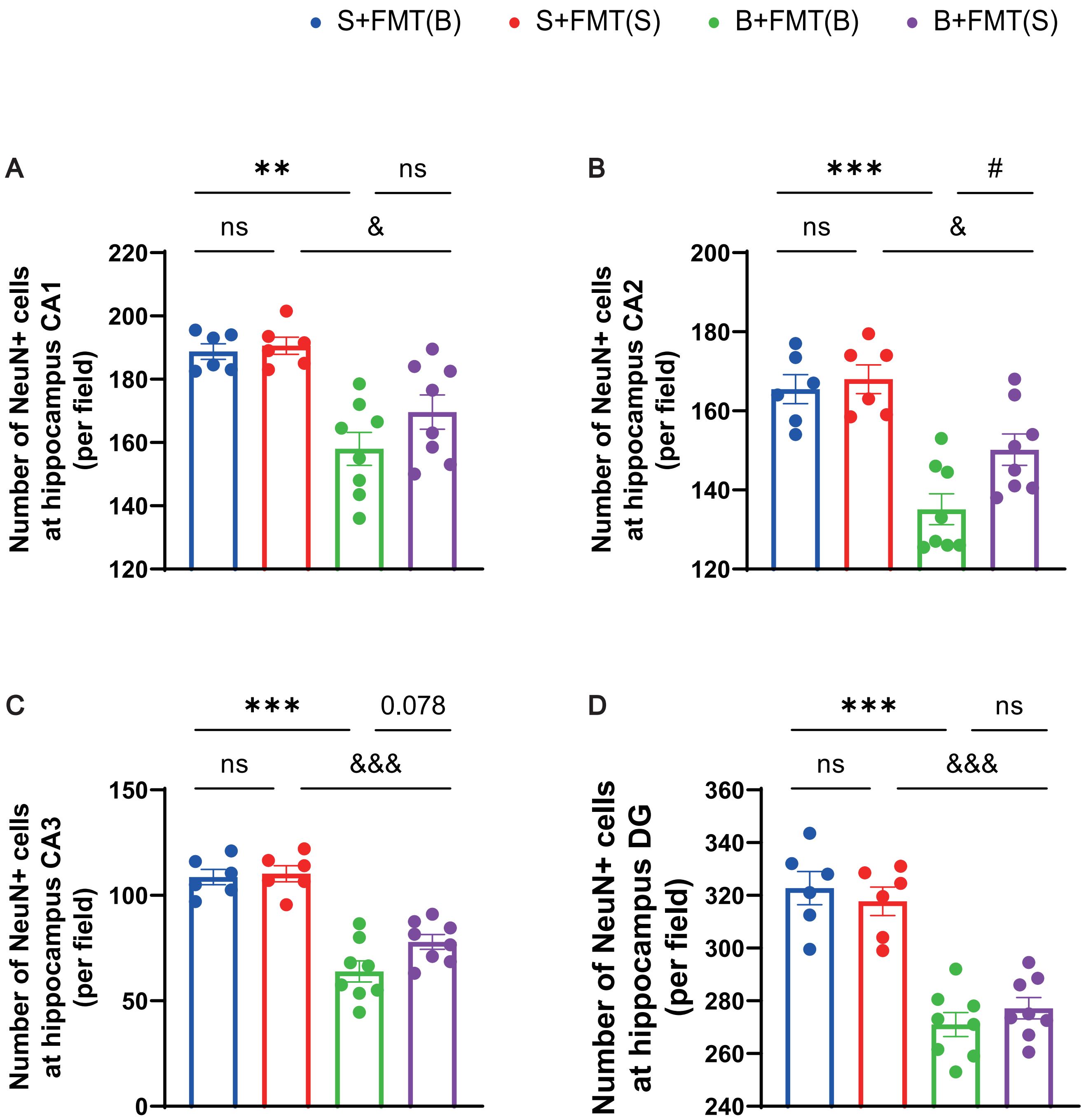

Fig. S9 Effects of FMT on the number of NeuN+ cells in different areas of the hippocampus, namaly (**A**) CA 1, (**B**) CA2, (**C**) CA3, and (**D**) DG (n = 6 S+FMT(B) and S+FMT(S), n = 8 B+FMT(B) and B+FMT(S)). The data represent the mean ± SEM, $p < 0.05$ was set as the threshold for significance. ** $p < 0.01$, *** $p < 0.001$ compared to the S+FMT(B) group. & $p < 0.05$, &&& $p < 0.001$ compared to the S+FMT(S) group. # $p < 0.05$ compared to the B+FMT(B) group.

Fig. S10

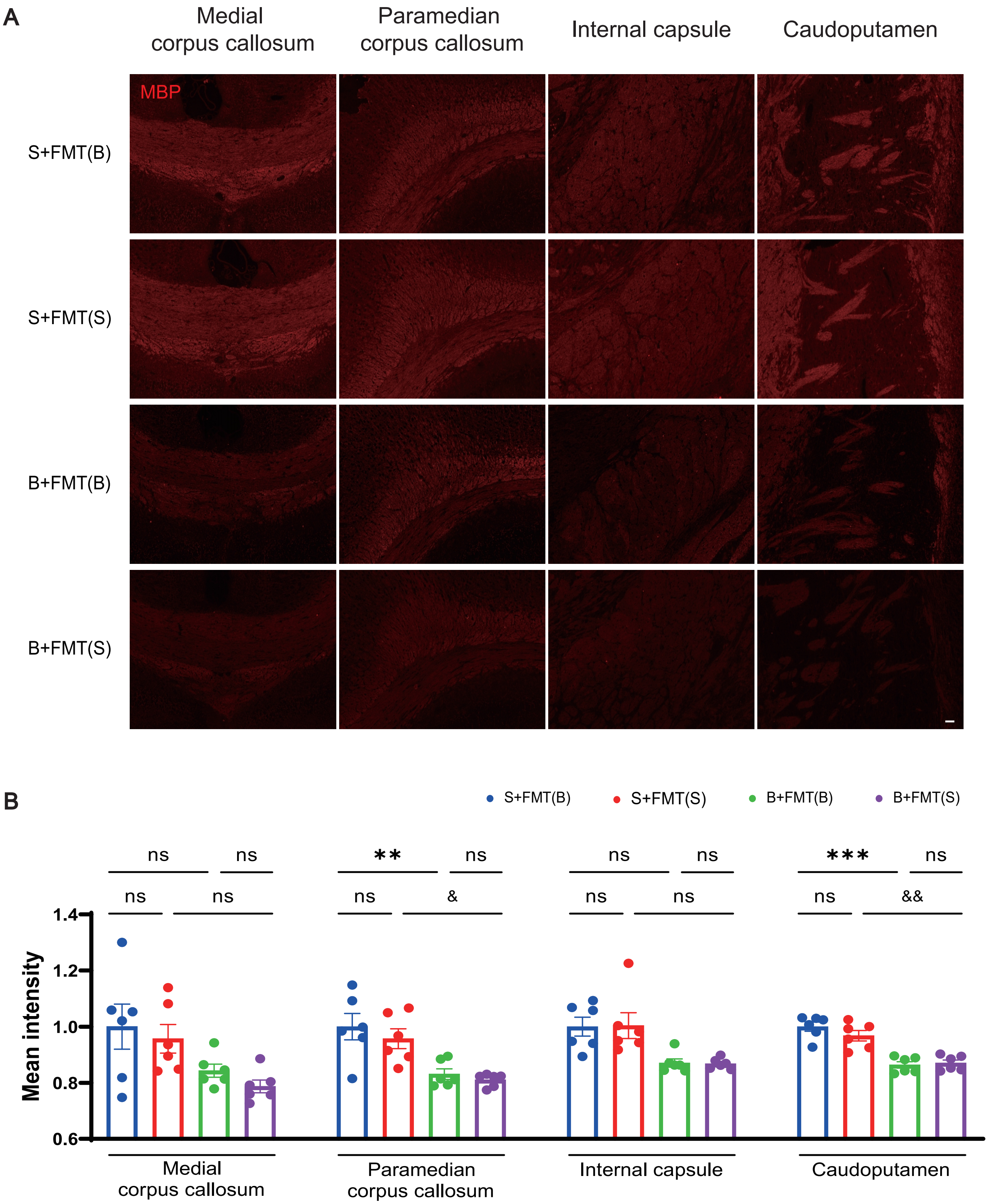

Fig. S10 Effects of FMT on white matter injury after BCCAO. **A** Representative immunofluorescence staining images of MBP in the medial CC, paramedian CC, IC, and caudoputamen regions. Scale bar, 50 μm. **B** Bar plots showing the mean fluorescent intensity (n = 6/group). The data represent the mean ± SEM, $p < 0.05$ was set as the threshold for significance. ** $p < 0.01$, *** $p < 0.001$ compared to the S+FMT(B) group. & $p < 0.05$, && $p < 0.01$ compared to the S+FMT(S) group.

Fig. S11

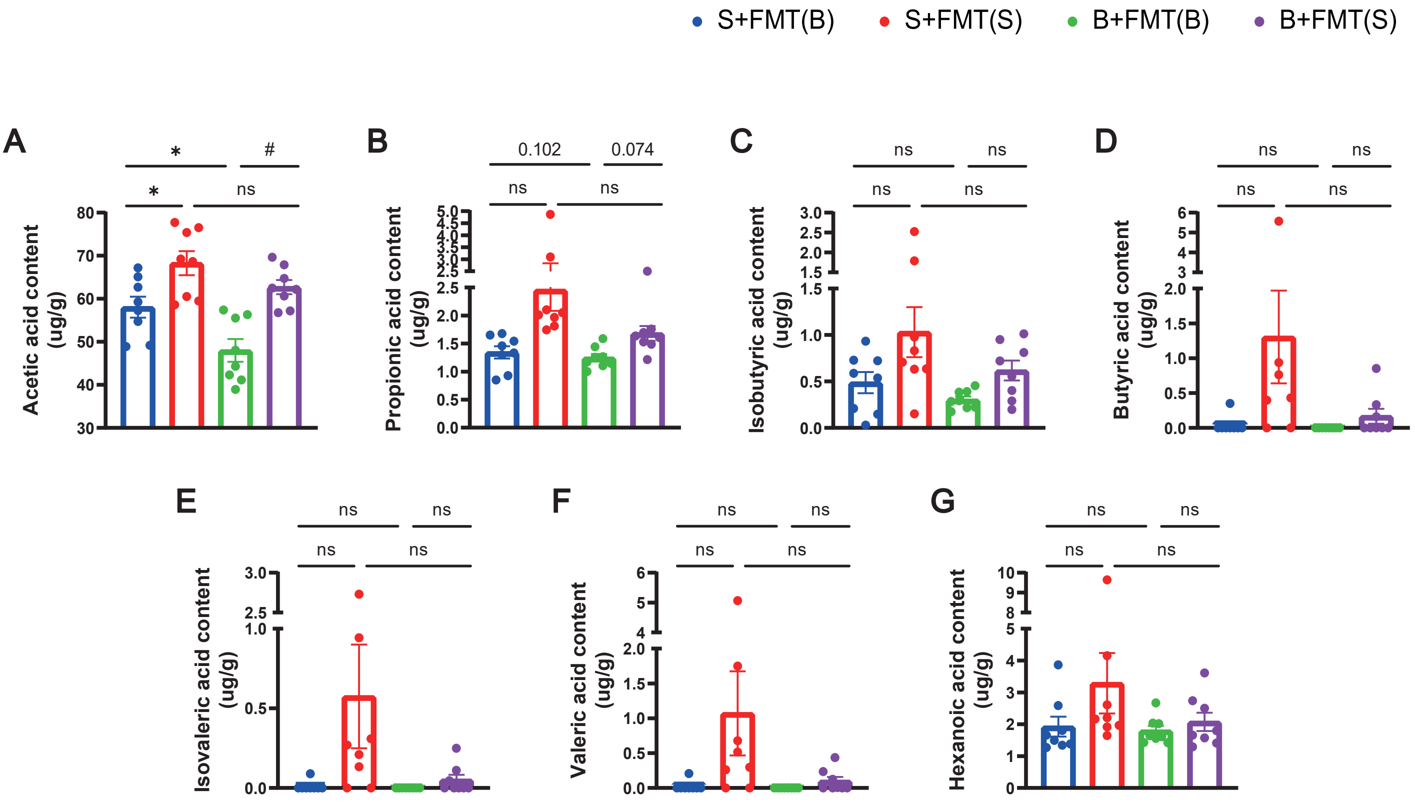

Fig. S11 Effects of FMT on SCFA levels in the hippocampus of recipient rats, including acetic acid (**A**), propionic acid (**B**), isobutyric acid (**C**), butyric acid (**D**), isovaleric acid (**E**), valeric acid (**F**), and hexanoic acid (G) (n = 8/group). The data represent the mean ± SEM, $p < 0.05$ was set as the threshold for significance. * $p < 0.05$ compared to the S+FMT(B) group. # $p < 0.05$ compared to the B+FMT(B) group.

Fig. S12

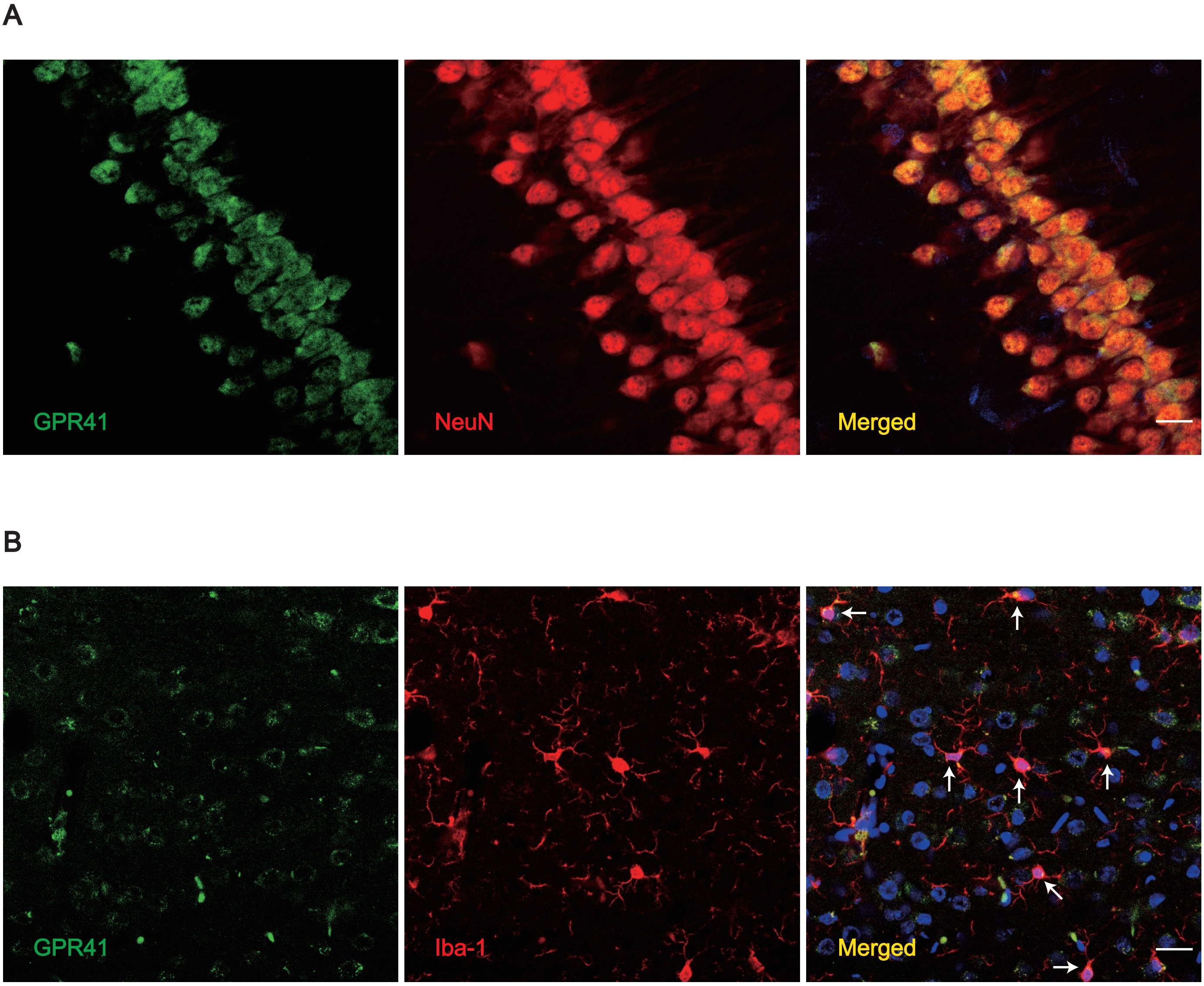

Fig. S12 Immunofluorescence staining of the GPR41-a receptor of SCFAs with hippocampal neurons (**A**) and microglia (**B**). Arrows indicate the expression of GPR-41 on microglia. Scale bar, 20 μm.

Fig. S13

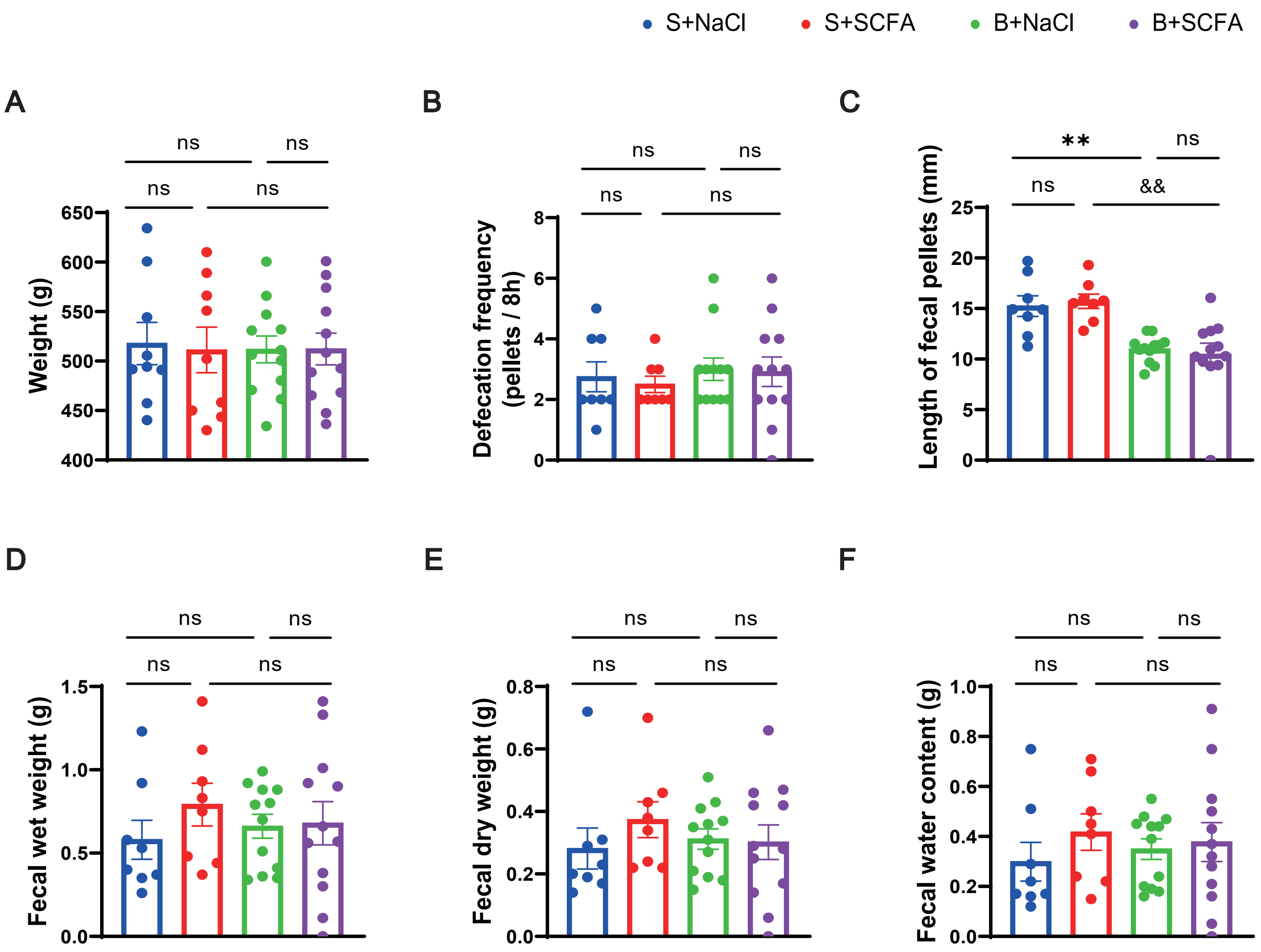

Fig. S13 Effects of long-term SCFA supplementation on body weight (**A**), defecation frequency (**B**), length of fecal pellets (**C**), fecal wet weight (**D**), fecal dry weight (**E**), and fecal water content (**F**) in rats (n = 8 S+NaCl and S+SCFA, n = 12 B+NaCl and B+SCFA). S+NaCl, sham rats treated with sodium chloride (NaCl). S+SCFA, sham rats that received short chain fatty acids (SCFAs) (acetate, propionate and butyrate) supplementation. B+NaCl, BCCAO rats treated with NaCl. B+SCFA, BCCAO rats received SCFAs supplementation. The data represent the mean ± SEM, $p < 0.05$ was set as the threshold for significance. ** $p < 0.01$ compared to S+NaCl group. && $p < 0.01$ compared to the S+SCFA group.

Fig. S14

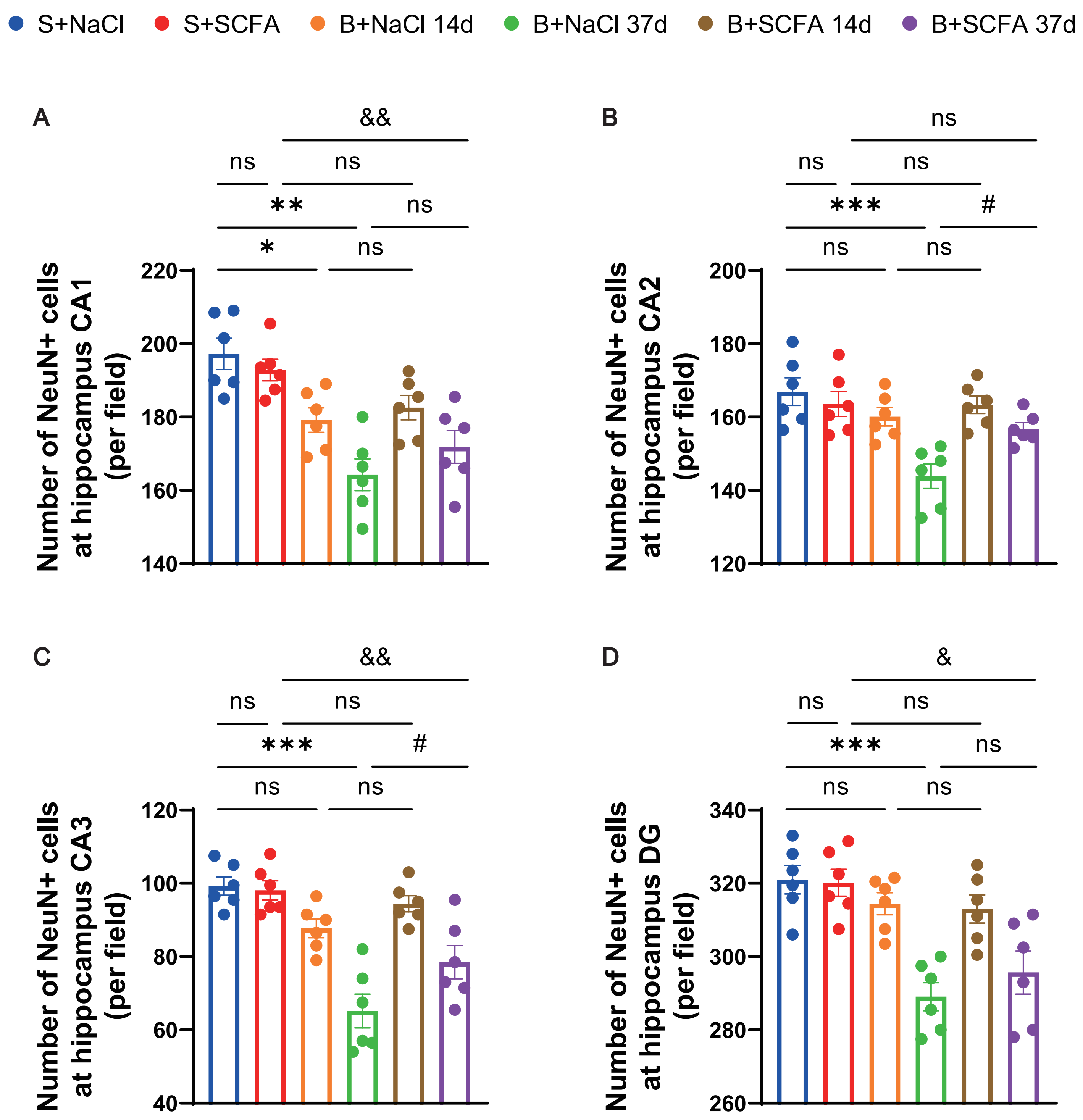

Fig. S14 Effects of long-term SCFA supplementation on the number of NeuN+ cells in different areas of the hippocampus, namaly (**A**) CA 1, (**B**) CA2, (**C**) CA3, and (**D**) DG (n = 6). The data represent the mean ± SEM, $p < 0.05$ was set as the threshold for significance. * $p < 0.05$, ** $p < 0.01$, *** $p < 0.001$ compared to the S+NaCl group. & $p < 0.05$, && $p < 0.01$ compared to the S+SCFA group. # $p < 0.05$ compared to the B+NaCl 37 d group.
