# Supplementary material for: The microbiota-gut-brain axis participates in chronic cerebral hypoperfusion by disrupting the metabolism of short-chain fatty acids
Source: Microbiome. 2022 Apr 17;10:62. doi: 10.1186/s40168-022-01255-6 (PMC9013454; doi:10.1186/s40168-022-01255-6)
Supplement: Supplementary file 5 — Additional file 4: Table S1. The primer sequences for real-time PCR. Table S2. The main mass spectrometry conditions for SCFA quantification. [file 40168_2022_1255_MOESM5_ESM.pdf]

Table S1 Primer sequences used in real-time PCR analysis

Primer	Sequence (5’ to 3’)
actin-rF	GTACAACCTTCTTGCAGCTCCTC
actin-rR	GTCCTTCTGACCCATACCCA
tumor necrosis factor alpha (*TNF-α*)-rF	GAAAAGCAAGCAACCAGCCA
TNF-α-rR	ACTGATGAGAGGGAGCCCAT
interleukin-1 beta (*IL-1β*)-rF	AGGCTGACAGACCCCAAAAG
IL-1β-rR	GGTCGTCATCATCCCACGAG
IL-6-rF	AGCGATGATGCACTGTCAGAA
IL-6-rR	GCATTGGAAGTTGGGGTAGGA
IL-4-rF	ACCCTGTTCTGCTTTCTC
IL-4-rR	GTTCTCCGTGGTGTTCCT
IL-10-rF	CCTCTGGATACAGCTGCGAC
IL-10-rR	AGTAGATGCCGGGTGGTTCA
mucin-2 glycoprotein (*Muc2*)-rF	GAGTTGTATGTGCTCGCCTG
Muc2-rR	CCACTGCTCACAGTCATTGGT
mucin-4 glycoprotein (*Muc4*)-rF	GACCACCAGCAGAACTCAAA
Muc4-rR	AGGATTGTGGGTGGTCTTCA
occludin-rF	GGACTGTTCAGAGCTCCGTC
occludin-rR	CACAGAGGTAGCACCACGTT
claudin-5-rF	CCTGCTAACCTGAAAGGGCA
claudin-5-rR	GGGACTGCTGGAATGAGACC
ZO-1-rF	ACAGCCAGCTCTTGGTCATC
ZO-1-rR	GTATGGTGGCTGCTCAAGGT
ionized calcium binding adaptor molecule 1 (*Iba1*)-rF	CGTCTGAGGAGCTATGAGCC
Iba1-rR	ATGGCAGATCTCTTGCCCAG
Fc RII/III receptor (*CD16*)-rF	TCCGTGGCAGTCTATGAGGA
CD16-rR	CAGATGGTGAGGTCGCAAGT
CD206-rF	AGTCTGCCTTAACCTGGCAC
CD206-rR	AGGCACATCACTTTCCGAGG

Table S2 The main mass spectrometry conditions

Settings	Parameters
Sample Volume	1μL
Front Inlet Mode	Split Mode(10:1)
Front Inlet Septum Purge Flow	3mL min^{-1}
Carrier Gas	Helium
Column	HP-FFAP（30m×250μm×0.25μm）
Column Flow	1mL min^{-1}
Oven Temperature Ramp	80°C for 1min; raised to 200°C at a rate of 10°C min^{-1}, hold on for 5min; raised to 240°C at a rate of 40°C min^{-1}, hold on for 1min
Front Injection Temperature	240°C
Transfer Line Temperature	240°C
Ion Source Temperature	230°C
Quad Temperature	150°C
Electron Energy	-70eV
Mass Range	m/z: 33-150
Solvent Delay	4min
